# Hominin Dispersal into the Nefud Desert and Middle Palaeolithic Settlement along the Jubbah Palaeolake, Northern Arabia

**DOI:** 10.1371/journal.pone.0049840

**Published:** 2012-11-19

**Authors:** Michael D. Petraglia, Abdullah Alsharekh, Paul Breeze, Chris Clarkson, Rémy Crassard, Nick A. Drake, Huw S. Groucutt, Richard Jennings, Adrian G. Parker, Ash Parton, Richard G. Roberts, Ceri Shipton, Carney Matheson, Abdulaziz al-Omari, Margaret-Ashley Veall

**Affiliations:** 1 School of Archaeology, Research Laboratory for Archaeology and the History of Art, University of Oxford, Oxford, United Kingdom; 2 Human Origins Program, Smithsonian Institution, Washington, D. C., United States of America; 3 Department of Archaeology, College of Tourism & Archaeology, King Saud University, Riyadh, Saudi Arabia; 4 Ministry of Higher Education, Riyadh, Saudi Arabia; 5 Department of Geography, King’s College London, London, United Kingdom; 6 School of Social Science, University of Queensland, Brisbane, Queensland, Australia; 7 CNRS, Maison de l’Orient et de la Méditerranée, Lyon, France; 8 Department of Archaeology, Connolly Building, University College Cork, Cork, Ireland; 9 Department of Anthropology and Geography, Oxford Brookes University, Oxford, United Kingdom; 10 Centre for Archaeological Science, School of Earth & Environmental Sciences, University of Wollongong, Wollongong, New South Wales, Australia; 11 Department of Anthropology, Paleo-DNA Laboratory, Lakehead University, Ontario, Canada; 12 The Saudi General Commission for Tourism and Antiquities, Taif Antiquities Office, Taif, Saudi Arabia; Illinois State University, United States of America

## Abstract

The Arabian Peninsula is a key region for understanding hominin dispersals and the effect of climate change on prehistoric demography, although little information on these topics is presently available owing to the poor preservation of archaeological sites in this desert environment. Here, we describe the discovery of three stratified and buried archaeological sites in the Nefud Desert, which includes the oldest dated occupation for the region. The stone tool assemblages are identified as a Middle Palaeolithic industry that includes Levallois manufacturing methods and the production of tools on flakes. Hominin occupations correspond with humid periods, particularly Marine Isotope Stages 7 and 5 of the Late Pleistocene. The Middle Palaeolithic occupations were situated along the Jubbah palaeolake-shores, in a grassland setting with some trees. Populations procured different raw materials across the lake region to manufacture stone tools, using the implements to process plants and animals. To reach the Jubbah palaeolake, Middle Palaeolithic populations travelled into the ameliorated Nefud Desert interior, possibly gaining access from multiple directions, either using routes from the north and west (the Levant and the Sinai), the north (the Mesopotamian plains and the Euphrates basin), or the east (the Persian Gulf). The Jubbah stone tool assemblages have their own suite of technological characters, but have types reminiscent of both African Middle Stone Age and Levantine Middle Palaeolithic industries. Comparative inter-regional analysis of core technology indicates morphological similarities with the Levantine Tabun C assemblage, associated with human fossils controversially identified as either Neanderthals or *Homo sapiens*.

## Introduction

Though climate change and its effect on people around the world today is receiving considerable attention from scholars, governments and the general public, we have little understanding about how past populations coped with and adjusted to marginal environments in many regions of the world. The vast desert regions of the Sahara and the Arabian Peninsula contain numerous archaeological sites, indicating that Pleistocene hominins penetrated these areas, living in more favourable habitats [1], [2]. However, until very recently, little information has been forthcoming about the age of the archaeological sites in arid zones, and hardly anything is known about the specific ecological settings that hominins occupied. This is an unfortunate situation as an understanding of the habitats in which hominins were living has implications about the peopling of Eurasia. The “greening” of desert areas would have attracted game and human populations, while conversely, increased aridity would have led to population contractions, and possibly population extinctions. Moreover, although geneticists and archaeologists have speculated about the generic routes of human movement outside of Africa [3], [4], [5], [6], [7], little systematic information has emerged about the nature and distribution of archaeological sites in the Arabian Peninsula, a critical geographic point in any discussion about out of Africa dispersal processes.

With respect to the palaeoenvironments of Arabia, cave speleothem records and lacustrine and sand dune deposits indicate that the region experienced dramatic climatic oscillations between wet and dry periods [8], [9]. Significant increases in precipitation have occurred during each interglacial period since at least Marine Isotope (MIS) 9 (330,000 years ago, or ka), with extensive speleothem, calcrete, alluvial fan and lacustrine records providing evidence of pluvial conditions [10], [11], [12], [13], [14]. Although northwestern regions of the Arabian Peninsula appear to have experienced some degree of increased humidity during glacial phases such as MIS 4 [15], [16], the vast majority of Arabia experienced increased dune mobility under hyper-arid conditions [17], [18], making large areas of Arabia uninhabitable.

Most archaeological sites in the Arabian Peninsula cannot be correlated with past environments as they are known from surface contexts, where chronometric dates and ecological information is not recoverable. Fortunately, several stratified archaeological sites have recently been investigated, making considerable contributions to our knowledge of Middle Palaeolithic occupation history. Middle Palaeolithic industries in Arabia are now known to date to MIS 5e [19] and later phases of phases of MIS 5 [19], [20], [21]. Middle Palaeolithic sites have not been dated to the arid period of MIS 4, although Shi’bat Dihya was occupied at 55 ka, at the beginning of MIS 3 [22]. The geographic location of the archaeological sites demonstrates human occupation in lacustrine [21] and riverine settings [20], [22]. The only published palaeobotanical study of a Middle Palaeolithic archaeological site in Arabia indicates occupation in a grassland setting with some woods at Jebel Qattar-1 [21], which will be further discussed below.

**Figure 1:**
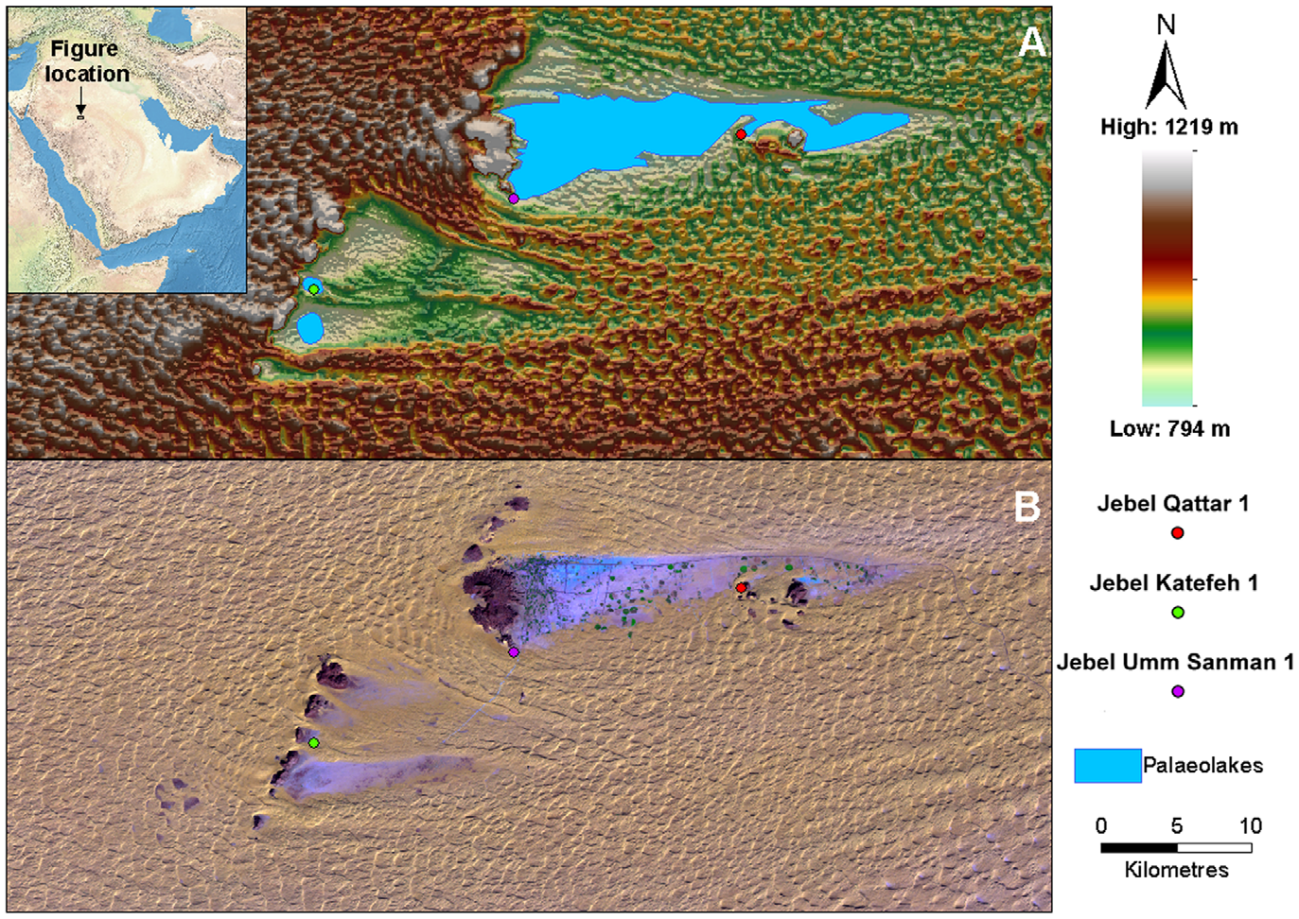
Location, topography and geomorphology of the Jubbah region. (A) Shuttle Radar Topography Mission Digital Elevation Model of the Jubbah region, overlain with interpreted palaeolake extents in blue. (B) Landsat ETM false color composite (bands 7, 4 and 1 on red, green and blue) of the Jubbah region, with exposed paleolake sediments appearing as the lightest blue shades, the rock outcrops as black, and the sand dunes light yellow. Both figures show the locations of the Middle Paleolithic sites identified during reconnaissance and discussed within the text.

Much information is yet to be gathered about Arabian palaeoenvironments and the nature of human occupations. In an attempt to help remedy this situation, we report here the presence of three stratified Middle Palaeolithic sites associated with the Jubbah palaeolake, a significant lake basin in the Nefud Desert of northern Saudi Arabia. We describe the ecological settings of the sites and their stone tool assemblages. We examine prehistoric technology and behaviour and discuss the implications of our findings relative to climate change and dispersal models.

**Figure 2:**
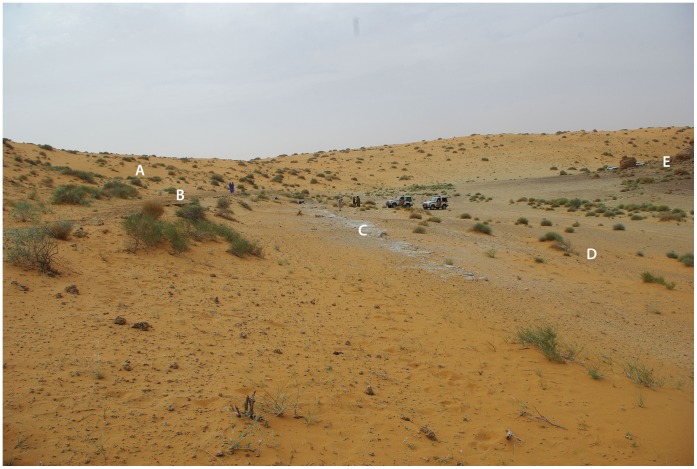
JQ-1 site looking west. A: Large sand dunes (MIS 4?); B: Upper palaesol (MIS 5a); C: Lower palaesol (MIS 5); D: Lower area; E: Base of Jebel Qattar footslope.

**Figure 3:**
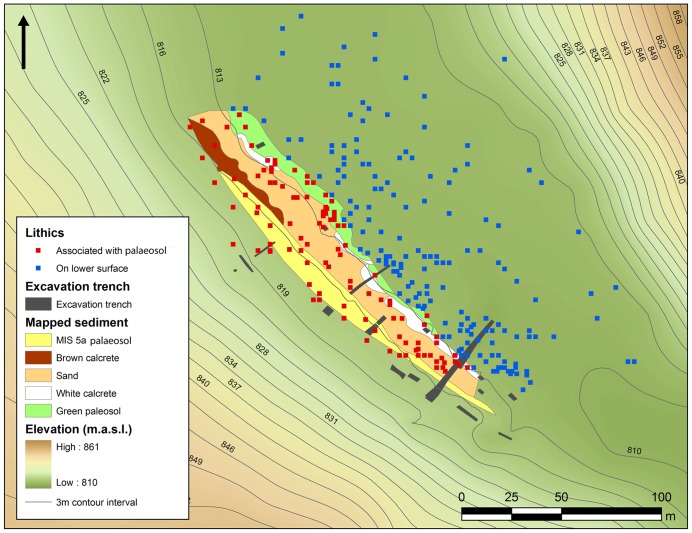
Topographic survey of surface artefacts and excavation trenches at JQ-1. Note distribution of artefacts on palaeosol and those on the slope immediately beneath it (in red) and those on lower surface (in blue). Sand dune to the left, Jebel Qattar to the right.

**Figure 4:**
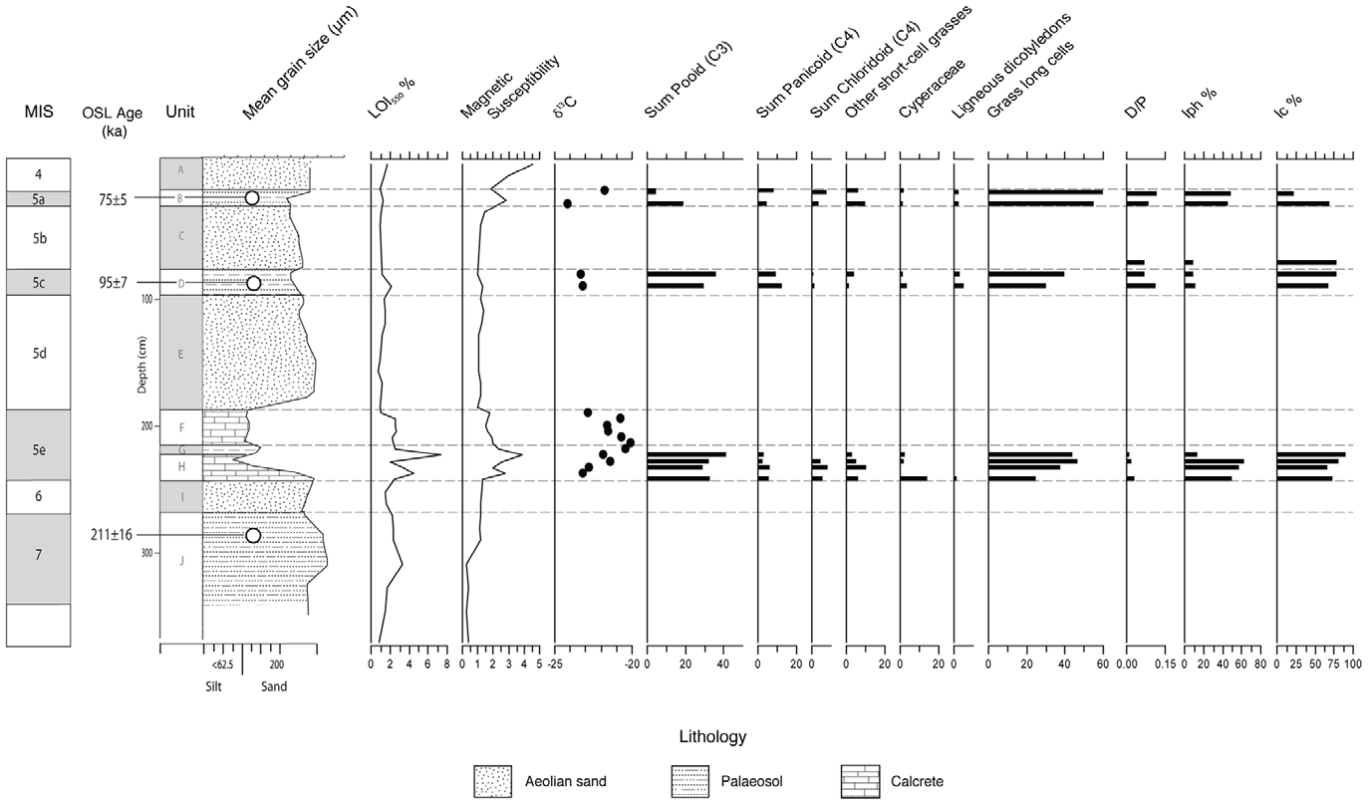
JQ-1 stratigraphic section. Stratigraphic log showing the locations of the palaeosols, calcretes and the OSL ages. The multiproxy record is also displayed, showing organic content (LOI_550_), magnetic susceptibility, δ^13^C and phytolith data. The bar to the left indicates that the OSL ages correspond to MIS 7 (211±16 ka), MIS 5c (95±7 ka), and MIS 5a (75±5 ka). The archaeological assemblage is associated with the upper palaeosol (Unit B, MIS 5a). The inter-stratifed sands between the palaeosols are interpreted as MIS 6, MIS 5d and MIS 5b deposits, and the overlying dune sands as MIS 4.

**Figure 5:**
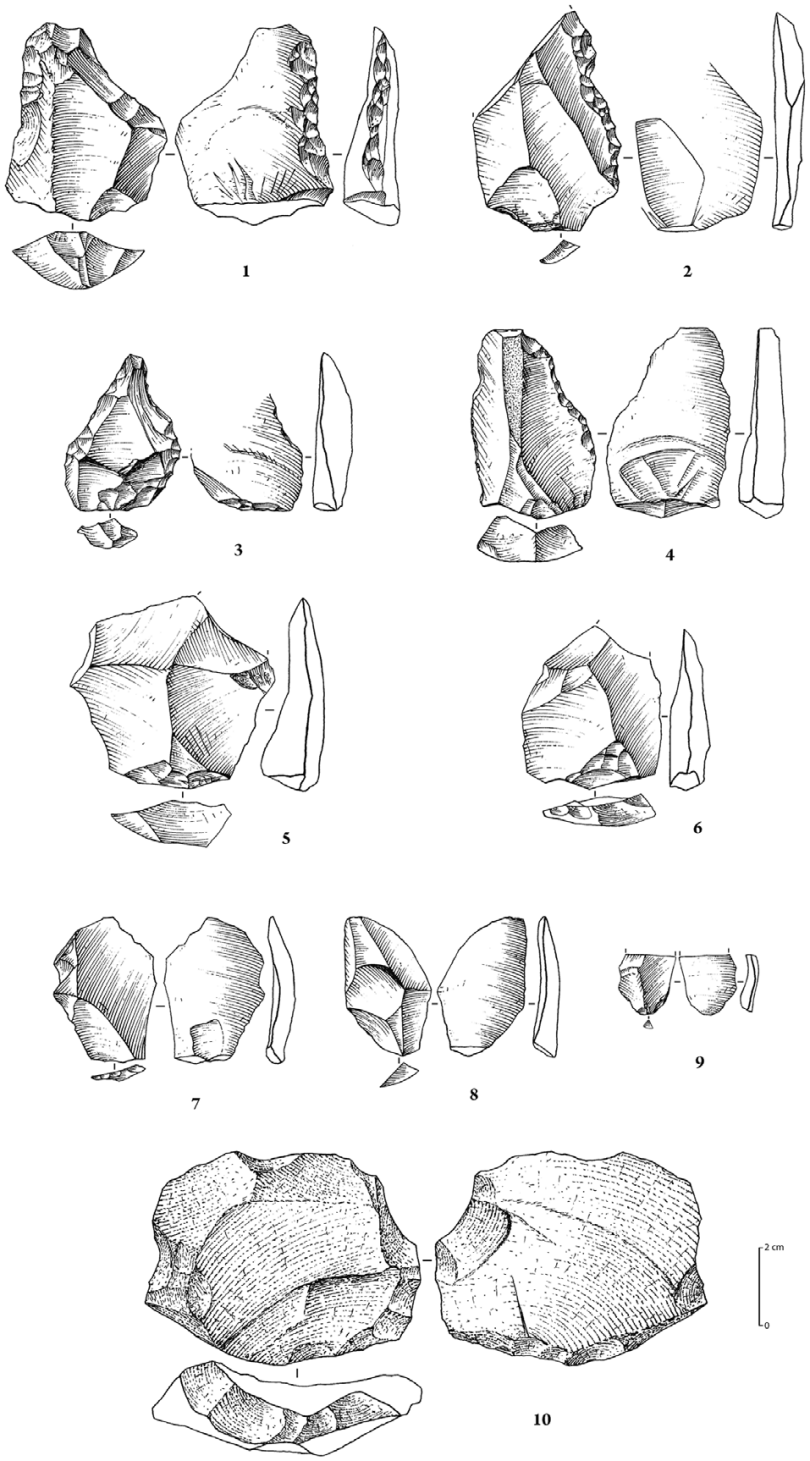
Stone tool assemblages from JQ-1. 1–4: retouched tools on flakes; 5: preferential Levallois flake with centripetal preparation; 6: recurrent centripetal Levallois flake. Artefacts from MIS 7 deposit: 7–8: (trench 16): flakes; 9 (trench 3): flake; 10 (trench 6): (preferential?) Levallois flake with faceted platform and centripetal dorsal scar pattern.

**Table 1:** Artefact class by site (no., percent).

	Debitage	Cores	Retouched	Total
**JQ-1 upper**	95 (81)	9 (9)	10 (10)	114
**JQ-1 lower**	28 (100)	0	0	28
**JKF-1**	1113 (91)	99 (8)	10 (1)	1222
**JSM-1**	74 (84)	10 (11)	4 (5)	88

**Table 2:** Raw material composition by site (no., percent).

	Quartzite+	Quartz	Rhyolite	Other++
**JQ-1 upper**	88 (77)	13 (12)	5 (4)	8 (7)
**JQ-1 lower**	14 (50)	5 (18)	2 (7)	7 (25)
**JKF-1**	930 (76)	277 (22)	9 (1)	7 (1)
**JSM-1**	81(92)*	4 (5)	0	3 (3)

+this calculation includes small numbers of quartzitic sandstone.

++primarily refers to fine grained siliceous materials (flint-like material).

* yellow fine-grained quartzite.

**Table 3:** Core types by site.

Core type	JQ-1+	JKF-1++ (quartz)	JKF-1 (quartzite)	JSM-1*	Total (%)
Single platform	1	24	3	–	28 (24)
Amorphous/other	–	11	7	–	18 (15)
Bidirectional	–	5	–	–	5 (4)
Radial	1	5	–	–	6 (5)
Discoidal	2	–	2	–	4 (3)
Fragment	–	4	4	2	10 (9)
Multiplatform	–	4	3	–	7 (6)
Recurrent centripetal Levallois	2	4	1	–	7 (6)
Recurrent unidirectional Levallois	–	–	4	–	4 (3)
Bidirectional Levallois	–	2	–	–	2 (2)
Unidirectional convergent Levallois	–	–	2	–	2 (2)
Preferential Levallois with centripetal preparation	2	2	7	5	16 (14)
Levallois preform	1	–	4	3	8 (7)
Total	9	61	37	10	117

+all cores produced from ferruginous quartzite, but for a single platform core from quartz.

++a single rhyolite core is a radial core (not included in tabulation).

* all cores produced from the yellow fine-grained quartzite.

**Table 4:** Debitage types by site.

Type	JQ-1	JKF-1	JSM-1	Total (%)
Flake	76	744	49	869 (68)
Blade	3	21	1	25 (2)
Core management elements	–	50	1	51 (4)
Chips/chunks/fragments	16	298	23	337 (26)
Total	95	1113	74	1282

**Table 5:** Retouched tool types by site.

Type	JQ-1	JKF-1	JSM-1
Side retouched flake	7	3	1
Side and end retouched flake	2	7	1
End retouched flake	–	–	1
Retouched point	1	–	–
Bifacial pieces	–	–	2

### Field Setting

The Jubbah palaeolake is located in the Nefud Desert of northern Arabia (Figure 1, Supporting Information S1). The desert floor of the Nefud is characterized by a network of relict lake and river systems [23], some of which have yielded Pleistocene fauna [24]. The activation of these wet systems relates to increased moisture from Mediterranean weather systems in the north [15] and perhaps, at times, from the Indian Ocean monsoon in the south [13], [25].

The exposed part of the Jubbah palaeolake lies downwind of large sandstone outcrops, particularly Jebel Umm Sanman to the west, which has diverted the westerly flow of sand around the outcrop, leaving a sand-free depression in its lee (Figure 1), measuring at least 20 km (east-west) and 4 km (north-south). The margins of the depression are concealed beneath aeolian sand to the north, east and south, with some dunes reaching heights of at least 60 m. The town of Jubbah is located within the western end of the basin, while central and eastern areas form an extensive agricultural belt fed by ground-water irrigation. Ground reconnaissance of several modern quarry exposures within the basin reveal a complex series of stratified aeolian and lacustrine beds. Modern water-wells in Jubbah and alongside Jebel Umm Sanman indicate that stratified sediments extend to a depth of at least 30 m, and include inter-digitated deposits of sands and silts and signs of a fluctuating water table. These deposits mostly underlay a radiocarbon age of 25,630±430 BP (Q-3117) [26], indicating ages for the deposits older than MIS 3, though caution is warranted given the sample was taken some time ago, and is a bulk age from humid soils. On the basis of the Digital Elevation Model (DEM) images, Landsat TM imagery and fieldwork, our expectation is that the northern depression filled with water during past humid periods. A second depression to the southwest is situated downwind of a series of five sandstone outcrops, and measures ca. 9 km (east-west)×6 km (north-south). This depression is at a higher elevation compared to the northern area, and ground reconnaissance revealed eroded surfaces with bare patches of sandstone bedrock, not apparent on the Landsat TM images. Within the southwestern depression, relict palaeolake sediments are exposed in mesa outcrops, with extensive evidence for deflation and erosional processes. On the basis of the DEM images, Landsat TM imagery and fieldwork, we have identified two smaller palaeolakes, one measuring 1 km^2^ (at Jebel Katefeh) and another in the extreme south, measuring 2.5 km^2^ (Figure 1).

**Figure 6:**
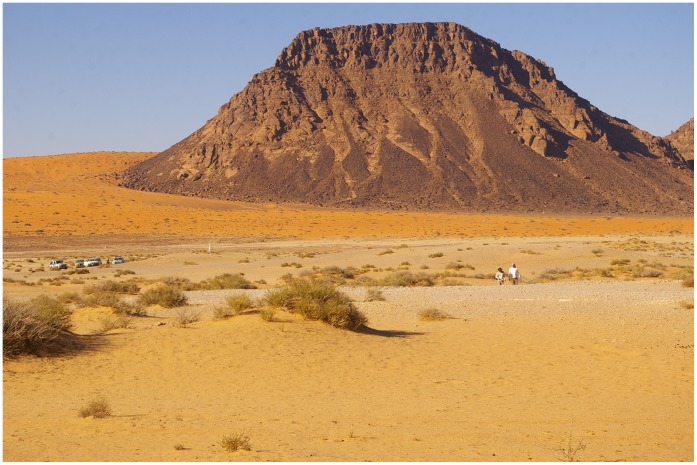
JKF-1 site looking west. Note the mound to the left. JKF-12 quartzite source is at the base of the jebel.

**Figure 7:**
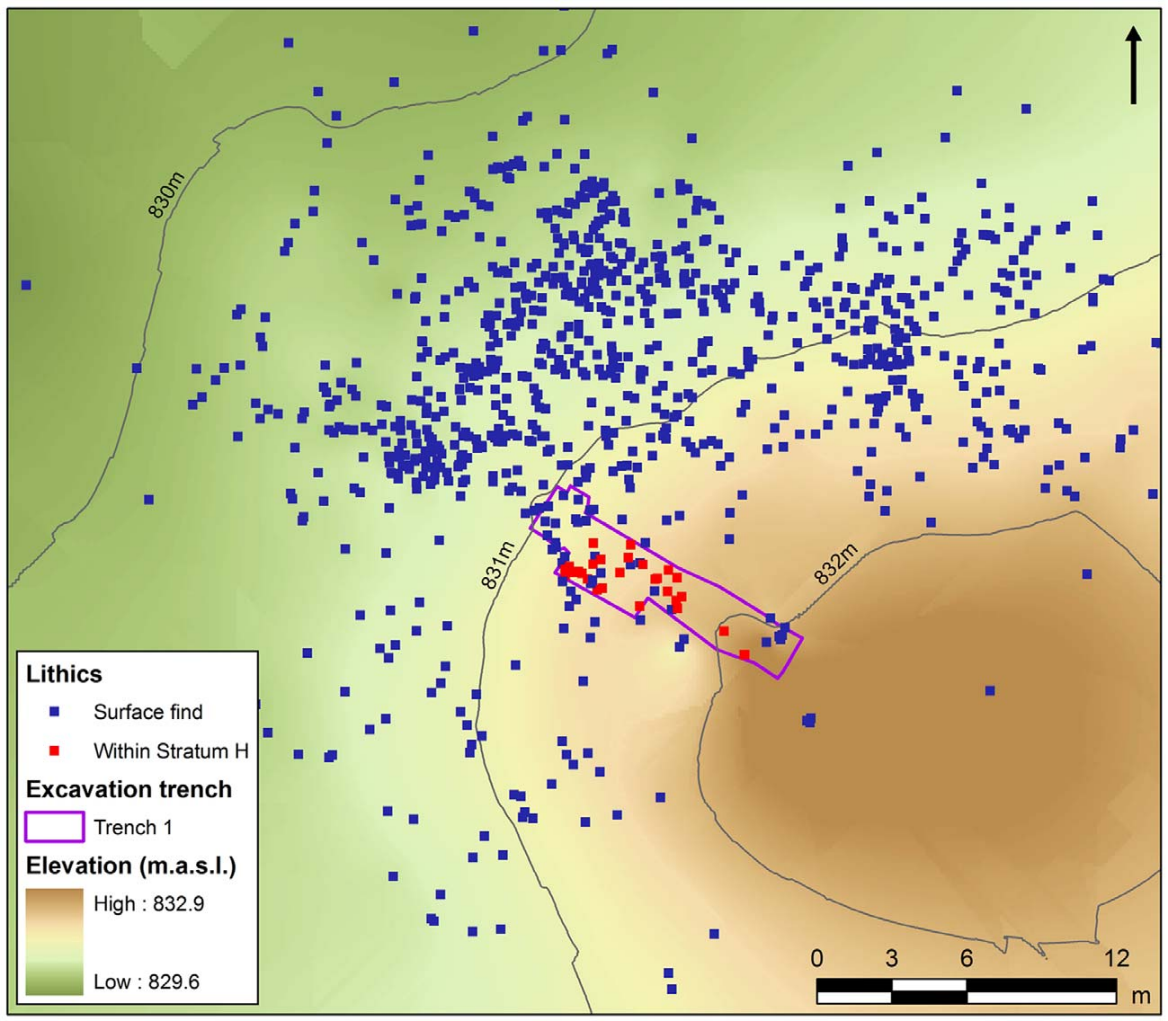
Topographic survey of surface artefacts at JKF-1 and location of trench. The location of trench 1 with buried artefacts (in red). Surface finds (in blue) mainly occur between the contour lines of 830–832 m, suggesting the presence of an extensively buried artefact surface. Refitted artefacts occur in buried and surface contexts, indicating a close relationship between surface and subsurface assemblages.

**Figure 8:**
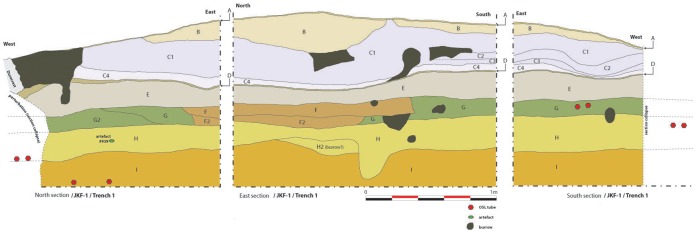
JKF-1 stratigraphic section. Stratigraphic levels A–J. Stratum H is the exclusive artefact horizon.

**Figure 9:**
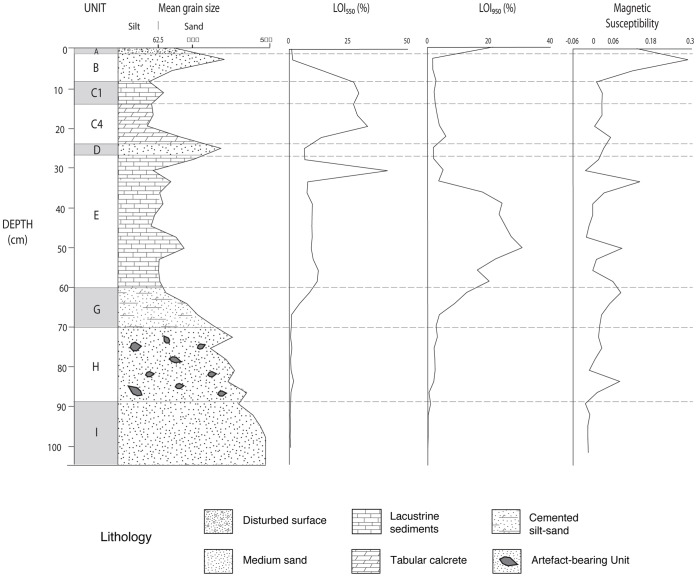
Stratigraphic log showing the locations of the palaeosols, calcretes and the OSL ages from the north section of Trench 1 at JKF-1. The multiproxy record is also displayed, showing organic content (LOI_550_), carbonate content (LOI_950_) and magnetic susceptibility.

**Figure 10:**
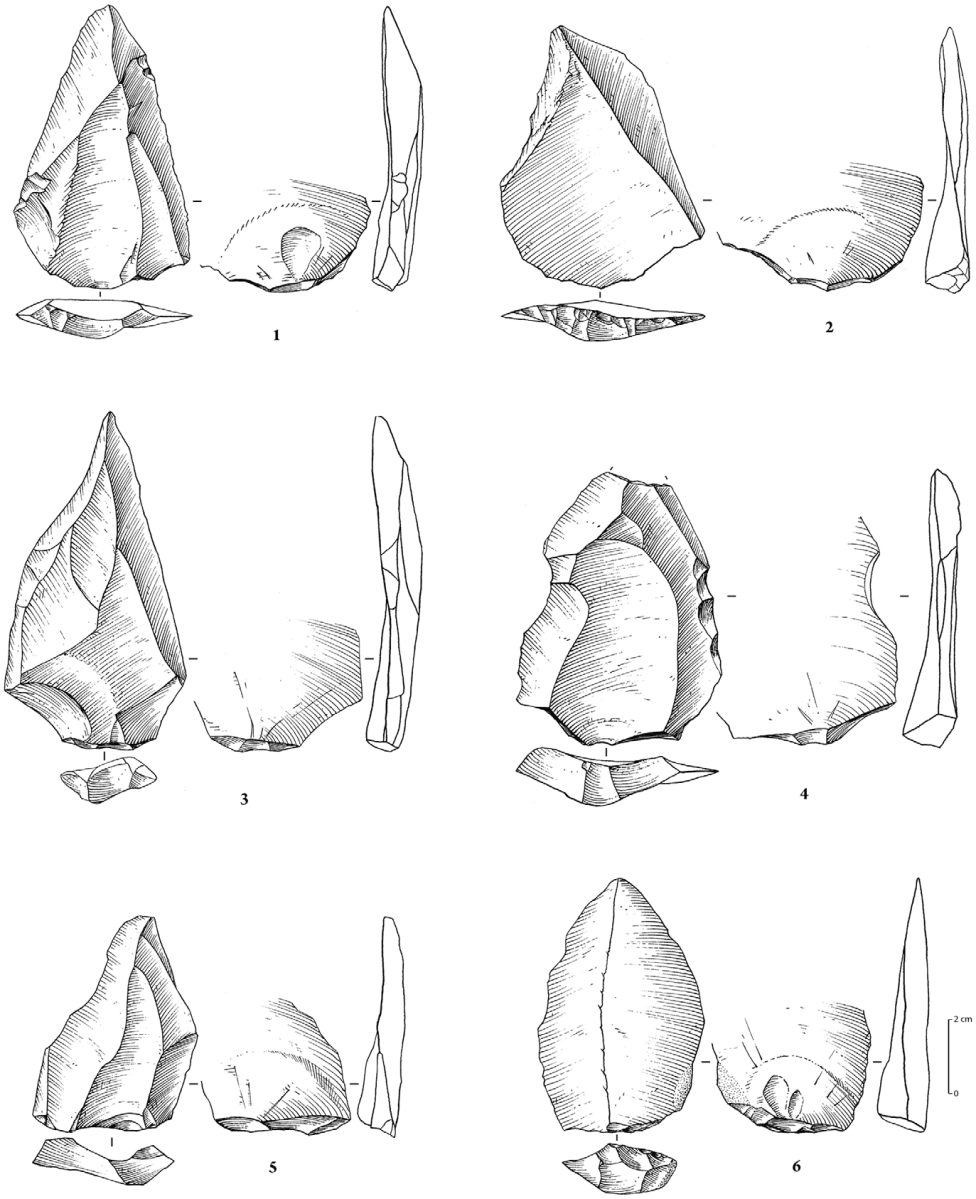
Stone tool assemblages from JKF-1. Ferruginous quartzite artefacts. 1 (no. 504, surface), 2 (no. S-76, stratified in stratum H), 3 (no. 513, surface): Levallois sub-triangular preferential flakes (Levallois “points”) with faceted platform and unidirectional convergent dorsal scar pattern; 4 (no. 921, surface): Levallois sub-triangular preferential flake with faceted platform and unidirectional convergent scar pattern, with some retouch on lateral mesial edge; 5 (no. 502, surface): Levallois sub-triangular preferential flake with dihedral platform and unidirectional convergent scar pattern; 6 (no. 521, surface): flake with faceted platform, produced on a ventral face of a flake.

**Figure 11:**
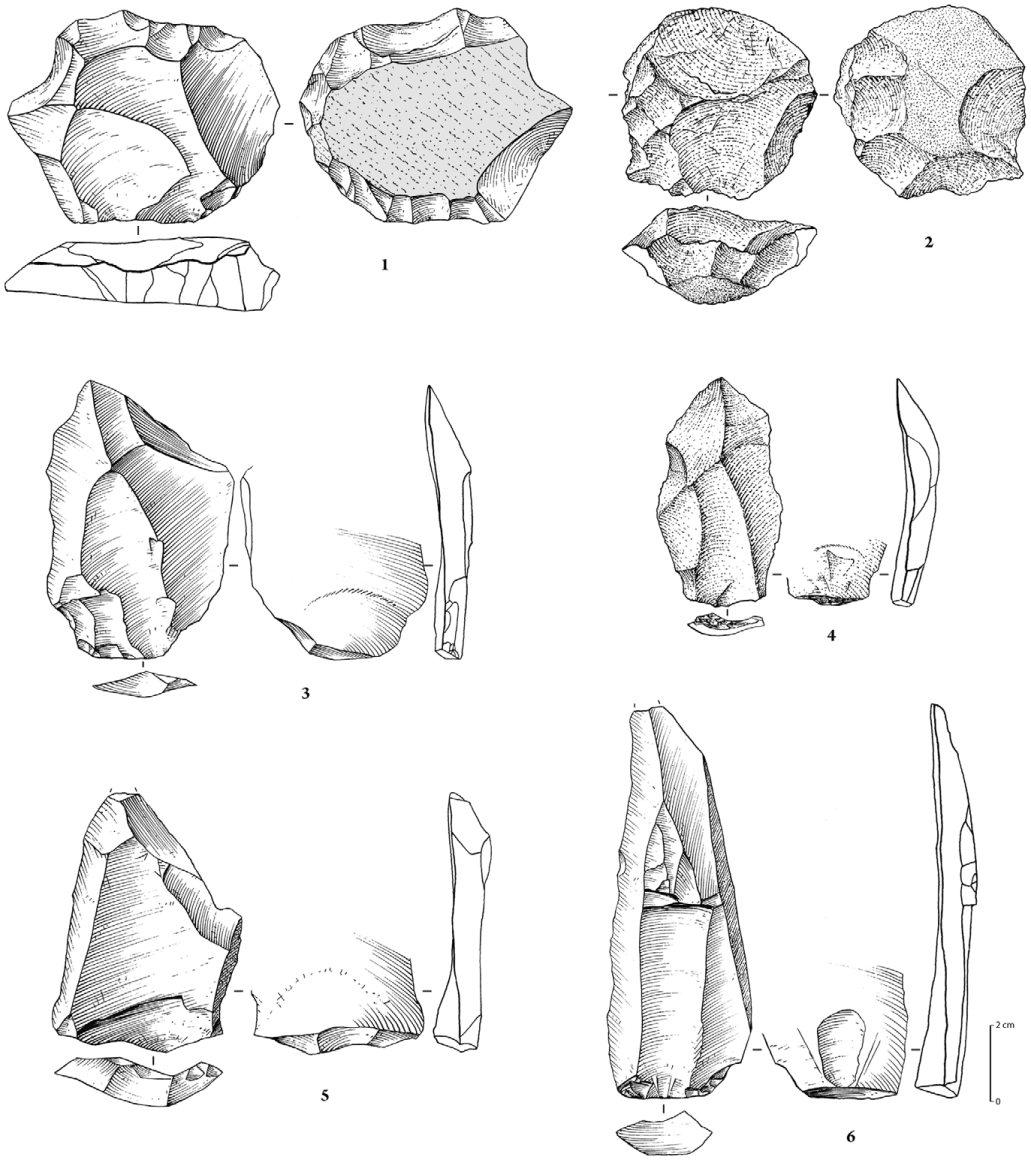
Ferruginous quartzite artefacts (except 2 and 4 on white quartz). 1 (no. 940, stratified in stratum H): Levallois core for preferential flake with centripetal preparation; 2 (units K9/L9, stratified in stratum H): recurrent centripetal Levallois core; 3 (no. S-20, Stratum H): preferential quadrangular Levallois flake with dihedral platform and unidirectional convergent dorsal scar pattern; 4 (no. 873, surface): Levallois elongated flake (preferential?) with faceted platform; 5 (no. 847, surface): Levallois flake with faceted platform and unidirectional crossed dorsal scar pattern; 6 (no. 209, surface): blade with plain platform and unidirectional convergent dorsal scar pattern.

**Table 6:** Biochemical results for the JKF-1 artefacts.

Artefact No.	Type	Residue	Location	Organic	Anthropogenic	Residue Analysis Results	Contamination
S-76	Levallois point	Yes	Lateral edge anddistal tip	Yes	Yes	Abundant evidence of plant (fattyacids, hydrocarbons, sterols) andanimal (fatty acids)	Fatty acids
504	Levallois point	Yes	Faceted platformand lateral edge anddistal tip	Yes	Yes	Evidence of plant and animal(fatty acids)	Fatty acids
S-20	Levallois preferential flake	Yes	Portion of facetedplatform and lowerlateral edge	Yes	Yes	Abundant evidence of plant - Brassicaceae(fatty acids, hydrocarbons, sterols)	Fatty acids, hydrocarbons
847	Levallois flake	Yes	Faceted platform andlower lateral edge withdamage	Yes	Yes	Evidence of plant (Brassicaceae)	Fatty acids
521	Flake	Yes	Faceted platform andlateral edge with slightnibbling	Yes	No	No evidence of authenticresidue	Fatty acids
209	Blade	Yes	Plain platform and twolower lateral edges	Yes	Indeterminate	Little evidence (Urea - proteinbreakdown or urine)	Fatty acids
873	Levallois preferential flake	Yes	Faceted platform andtwo lower lateral edges	Yes	Yes	Evidence of animal (fatty acids)	Fatty acids

## Results

The presence of Middle Palaeolithic hominins in northern Arabia is demonstrated by the occurrence of numerous surface archaeological localities [2], [27]. In the Nefud Desert, ‘Mousterian’ lithic assemblages were first reported from a factory/quarry site at Jubbah, near the summit of Jebel Umm Sanman and localities on the Jubbah palaeolake margins and floor [26]. The Jubbah artefact assemblages were found on the ground surface, as is commonly the case for other reported Palaeolithic occurrences in regions where erosion and deflation are often the dominant geomorphological processes. However, during our reconnaissance of Jubbah in March 2010, we identified a wealth of Palaeolithic occurrences along the edges of the palaeolake and the surrounding jebels. As reported below, we identified three buried and stratified archaeological sites.

**Figure 12:**
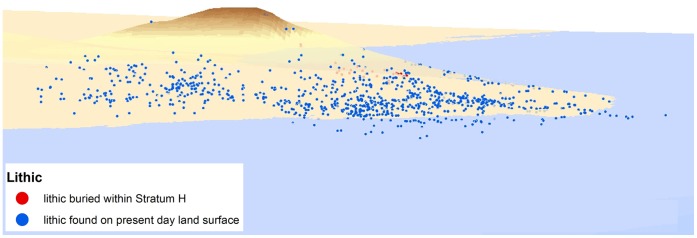
Proposed paleolake shoreline at JKF-1. Note that in situ artefacts occur in the middle of the mound. Artefacts occur on a palaeodune surface, situated above a lower-lying shallow lake.

**Figure 13:**
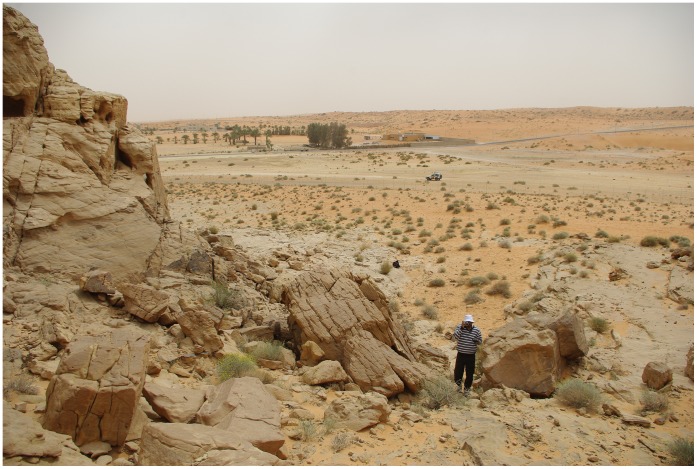
Location of yellow quartzite seam at JSM-1, looking northeast. The seam is at the base of the jebel hillslope. The Jubbah palaeolake bed is in the background (where trees are located). Dune surround the lakebed (on right of photograph, view southeast). The JSM-1 site is off of the photograph, to the right.

**Figure 14:**
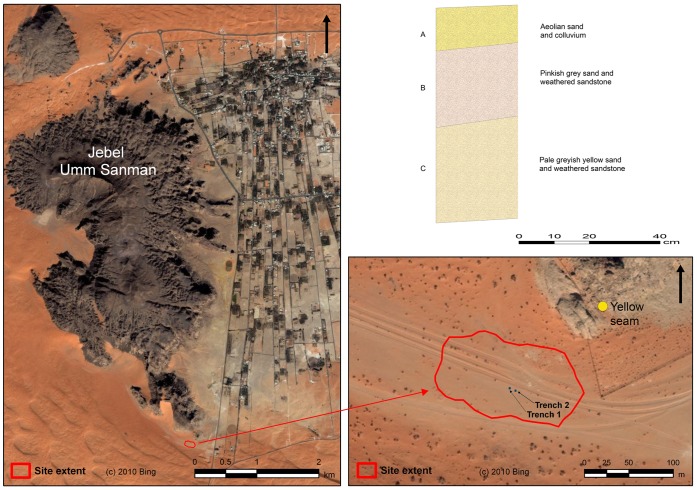
View of Jebel Umm Sanman and location of excavation units at JSM-1. The Jebel Umm Sanman is the largest jebel in the area. The modern town of Jubbah is developing on the former lake bed. Note the location of yellow quartzite seam relative to artefact finds.

**Figure 15:**
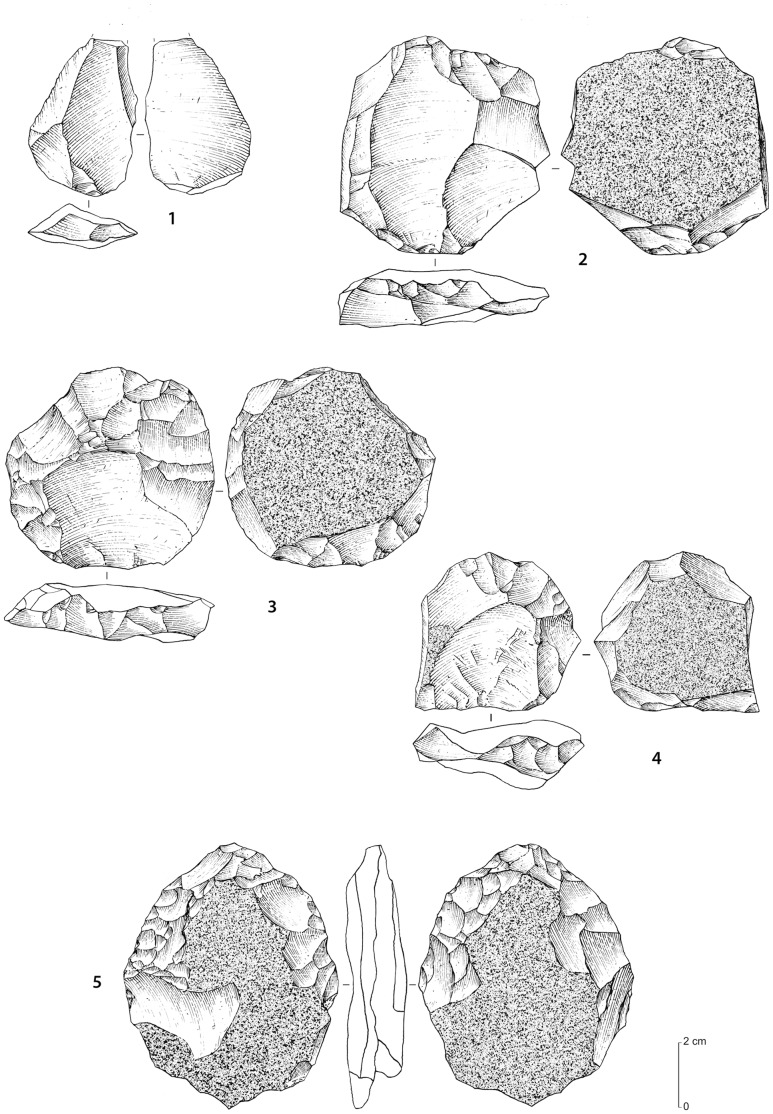
Stone tool assemblages from JSM-1. Yellow fine-grained quartzite artefacts. 1 (Trench 1, spit 2): Levallois flake from centripetal recurrent debitage, with dihedral platform; 2 (no. 81, surface), 3 (no. 1, surface), 4 (no. 84, surface): Levallois core for preferential flake removal, with centripetal preparation; 5 (no. 82, surface): biface on a flat tabular slab.

**Figure 16:**
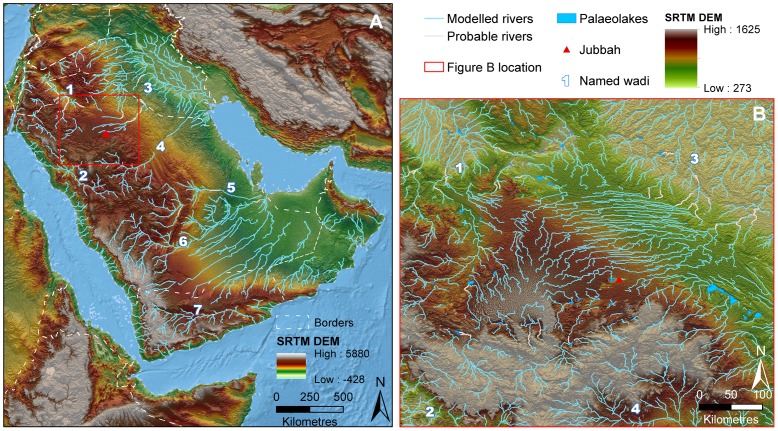
Palaeodrainage networks of Arabia and the An Nefud Desert. Key wadis are numbered and international borders displayed by dashed lines. Drainage network data (blue) is superimposed upon SRTM V.4 elevation data, overlain upon Natural Earth 2 data for the oceanic regions. Interpreted channel connections potentially active during recent wet phases are marked in grey. A: Simplified principal drainage networks for the Arabian peninsula. B: Detailed palaeodrainage networks for the Nefud region around the Jubbah sites, with palaeolakes identified through remote sensing displayed in light blue. Numbered widyan/rivers: 1- Wadi as Sirhan, 2-Wadi al Hamd, 3- Euphrates and widyan draining into the Euphrates basin, 4-Wadi al Batin, 5-Wadi Sabha, 6- Wadi ad Dawasir, 7- Wadi Hadramawt.

**Figure 17:**
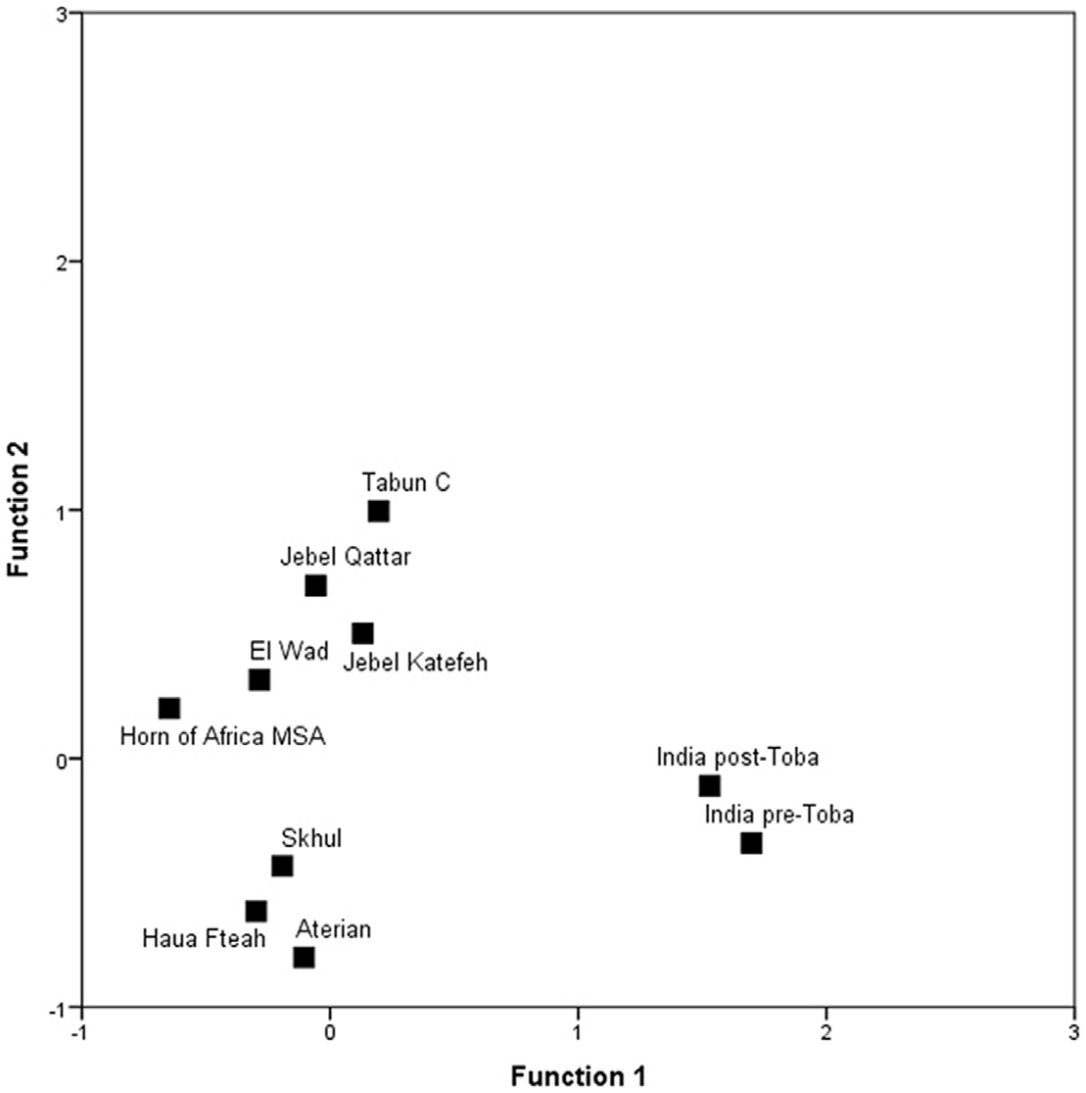
Discriminant Function Analysis of global patterns in core technology and placement of Jubbah artefacts. Note that the Jubbah artefacts are most similar to the Levantine Tabun C assemblage.

**Table 7:** Structure matrix for Discriminant Functions Analysis of Middle Palaeolithic cores.

Attribute	Function 1	Function 2
Proportion of Blades	0.399	0.625
Average Platform Angle	0.301	−0.159
Relative thickness	0.3	0.039
Scar Pattern Index	−0.276	−0.408
Intersection Height	0.231	−0.07
Elongation	0.066	0.319
Contracting/Expanding	0.06	0.088
Distal Curvature	0.024	−0.003
Largest Scar Proportion	0.006	−0.102

### Jebel Qattar

Jebel Qattar 1 (JQ-1) (28°00′53.20 N, 41°03′35.94 E) is located just south of a sandstone jebel and close to the southern edge of the exposed palaeolake basin (Figures 1–2). Jebel Qattar reaches a maximum height of 905 m above sea level, and the site of JQ-1 is situated on exposed sedimentary deposits at 816 m. The sedimentary deposits are adjacent to and overlain by a 30-m high sand dune. In 2010, a 1-m wide trench was excavated to 3 m in depth [21]. Fieldwork in 2011 primarily consisted of the expansion and deepening of Trench 1 and the excavation of 18 additional trenches across the site to expose subsurface deposits and identify archaeological horizons (Figure 3). The sediments included calcretes and weakly developed/incipient palaeosols within sandy deposits (Figure 4).

Loss on ignition organic content (LOI_550_) and carbonate content (LOI_950_) were conducted following the standard procedures [28]. To determine grain size, sediment samples were gently disaggregated in de-ionised water and analysed using a Malvern Mastersizer 2000. Mass specific, low frequency magnetic susceptibility measurements (χlf) were obtained from each sediment sample using a Bartington MS2 meter with an MS2C sensor at 0.1 SI sensitivity [29]. We used optically stimulated luminescence (OSL) dating to obtain burial ages for sand-sized grains of quartz. Three OSL ages were obtained from a 3.65 m section (Figure 4), including ages of 211±16 ka (lower), 95±7 ka (middle) and 75±5 ka (upper) [21]. The OSL ages are in correct stratigraphic order and provide important information regarding the timing of climatic amelioration within the Nefud.

The sedimentary sequence at JQ-1 is comprised of a stratified sequence (Figure 4) of aeolian, palaeosol and calcrete deposits (Units A–J). The aeolian component comprises moderately sorted medium sands with generally muted LOI_550_ and magnetic susceptibility values, reflecting increases in regional aridity. Given the stratigraphic position of aeolian sand units in relation to retrieved OSL ages, it is likely that these units represent MIS 4, MIS 5 b and d and MIS 6. Increases in regional humidity are indicated by the formation of incipient palaeosols and calcretes. An OSL age of 211±16 ka was determined for the lowermost palaeosol (Unit J), which reflects a period of increased humidity during MIS 7 and displays increases in both magnetic susceptibility and sediment organic content (LOI_550_). This is in agreement with other records from within Arabia [10], [11], [12], [13], which indicate that an increase in precipitation associated with both Mediterranean and monsoon atmospheric systems occurred at this time.

The MIS 7 palaeosol is overlain by aeolian sands and an interstratified calcrete/palaeosol deposit which, due to its stratigraphic position, we attribute to MIS 5e. The occurrence of dramatically increased humidity across Arabia during this period is well documented within a variety of palaeoclimatic records [9], [10], [12], [13], [16], which indicate that precipitation levels may have exceeded all other interglacials from MIS 9 through to MIS 1 [12]. MIS 5e deposits at JQ-1 are characterized by comparatively sharp increases in organic content and magnetic values, the latter reflecting increased fluvial influx (and watershed erosion). This is supported by positive shifts in δ^13^C, which reflect a contribution of terrestrially derived plant debris from watershed vegetation. At the base of this calcrete, phytolith and δ^13^C data reveal a C3 Pooid-dominated grassland with some C4 Panicoid and Chloridoid elements. Phytoliths are mostly absent during the later stages of MIS 5e, due to diagentic dissolution, which is marked by a brief phase of incipient palaeosol formation and peak δ^13^C values.

The MIS 5c paleosol (Unit D–95±7 ka) consists of poorly sorted silts and medium-grained sands, with calcareous rhizolith casts and phytoliths which indicate C3 Pooid-dominated grassland with some tree cover and C4 Panicoid, but little C4 Chloridoid grass cover; this interpretation is corroborated by δ^13^C values of 23%. The uppermost palaeosol (Unit B) was formed during MIS 5a (75±5 ka) with phytolith evidence indicating that at this time, the landscape was dominated by a mix of C3 Pooid and C4 Panicoid and Chloridoid grassland types, with some trees. A shift to drier conditions during MIS 5a is indicated by a slight increase in C4 Chloridoid grasses coupled with a 2% shift in δ^13^C values, however, the vegetation is never dominated by C4 flora. The MIS 5a palaeosol is overlain by massive sands, representing a major change in the depositional regime, which we interpret as evidence for climatic desiccation at the start of the last glacial period (MIS 4), which would be consistent with other dated records for the region [13], [16], [18], [30].

In arid/semi-arid regions such as the Jubbah basin, increased wetness during humid phases such as MIS 5e, coupled with increased vegetation cover and landscape stability, was crucial in promoting palaeosol and calcrete formation at JQ-1. The topographic position of the MIS 5e calcretes discussed here, cannot preclude either groundwater or palustrine influence as an agent for their formation, however, their presence is an important indicator for a substantial increase in regional humidity at this time. The evidence from JQ-1 for a significantly wetter climate during MIS 5e is consistent with other palaeoclimatic records from Arabia at this time, and indicates that lake formation within the Jubbah basin likely achieved its maximum extent during MIS 5e and covered a smaller area during other pluvials such as MIS 7, MIS 5a and c. These periods, however, would have been substantially more humid than today, as evidenced by extensive palaeosol formation. It is suggested that these palaeosols would have formed along lake margins, providing an attractive setting for Middle Palaeolithic populations. Additionally, tufa deposits 300 m east of JQ-1, at the base of the jebel, preserve abundant rhizoliths further indicating that freshwater sources were widely available during humid phases.

A total of 518 lithic artefacts were recovered in association with the raised MIS 5a palaeosol and the lower-lying surface extending northwards (Figure 3). The location of each of the artefacts recovered on the palaeosol in 2010 was recorded using a hand-held GPS whereas the remainder of the artefacts recovered in the 2011 season were recorded using a total station. A total of 114 artefacts are distributed on and immediately beneath the MIS 5a deposit, over an area measuring 150×25 m^2^ (Figure 3). Excavation at the contact between the MIS 5a palaeosol and the sand dune recovered several buried artefacts in a spatially limited and thin deposit of compact pale grey and orange silty sand. The palaeosol was not a continuous surface beneath the sand dune, however, hence buried artefacts could not be traced across this surface. The artefacts exposed on the palaeosol were mostly fresh to moderately rounded, without signs that they were exposed to the surface for a long period of time. Although the facets and edges of some artefacts showed rounding, we expect that some of this modification resulted from in situ chemical alterations rather than from wind abrasion alone. The 404 lithic artefacts on the lower surface were widely distributed over an area measuring 250×75 m^2^. Though debitage (n = 344), cores (n = 39) and retouched pieces (n = 21) were recovered in the lower area, the area experiences surface runoff, resulting in a mix of Middle Palaeolithic artefacts with younger materials, thus precluding detailed technological analysis.

The 114 artefacts recovered in association with the MIS 5a palaeosol consisted of 95 pieces of debitage (81%), 9 cores (9%) and 10 retouched tools (10%) (Table 1). Ferruginous quartzite (77%) dominates the assemblage, followed by quartz (12%), rhyolite (4%) and other materials (7%) (Table 2). The cores are generally small, and include discoidal and preferential centripetal and recurrent centripetal Levallois types (Table 3). The majority of the debitage is composed of flakes (n = 76), with recovery of only 3 blades (Table 4). Faceting occurs on 19% of the flake blanks. The 10 retouched tools include pieces that were informally retouched along their end- and side-margins (Table 5), typically retouched along a single lateral margin (Figure 5). Among the tools are a pseudo-Levallois point and a retouched point with ventral retouch along both lateral margins [21]. The overall manufacturing techniques are the production of short flakes from discoidal cores, flakes from centripetal recurrent Levallois cores with faceted preparation, and preferential flakes from Levallois cores with centripetal preparation.

A total of 28 pieces of debitage was recovered from the MIS 7 upper paleosol (Table 1). The artefacts are produced from quartzite (50%), quartz (18%), rhyolite (7%) and other materials (25%) (Table 2). A chi-squared test showed this raw material distribution to be different from those of the upper layer at the P<0.005 level. Two of the artefacts indicate the use of the Levallois flaking method, such as a thick and wide faceted flake with a centripetal dorsal scar pattern (Figure 5). The artefacts are mostly small (<2 cm), differing in size and raw materials in comparison with those recovered in association with the MIS 5a palaeosol.

Around the base of Jebel Qattar, seams of ferruginous quartzite are found in the sandstone, and quartzite also occurs as weathered clasts at the base of the Jebel’s slope. The weathered clasts are small and hard, with many natural inclusions. The generally small size of the JQ-1 cores therefore relates to the size and quality of this raw material. Within the sandstone are small quartz pebbles, which weather to form common clasts in spatially extensive gravel sheets in the lower area. Given the local occurrence of quartzite and quartz at JQ-1, the site to source distance for the transport is perhaps no greater than 100 m. On the other hand, the recovery of small numbers of rhyolite and flint artefacts implies long-distance transport, since no volcanic and siliceous sources have yet been identified at Jubbah. It is also possible that these materials were procured locally from unknown stream sources that included secondary materials.

### Jebel Katefeh

Jebel Katefeh 1 (JKF-1) (27°55′18.64 N, 40°48′18.14 E) is situated in a basin ca. 800 m east of the Jebel. A remnant mesa of relict aeolian sediments overlain by lacustrine silts, and capped by a calcrete deposit, measures ca. 40 m in diameter, rising to a height of 3 m above the current ground surface (Figure 6). Artefacts are distributed on the surface of the mound and its flanks, though a distinct concentration is present from about halfway down the mound on its western side (i.e., between 830–32 m contours) (Figure 7). A comprehensive surface collection of the lithics across the mound was conducted, and with each of the 923 artefacts piece-plotted using a total station. The surface artefacts showed weathering but they were in generally good condition, suggesting that they were not exposed to the surface for a long period of time.

A trench measuring 2 m in width and 12 m in length was excavated, beginning at the top of the mound and extending downslope to the west. Nine stratigraphic layers were differentiated in the trench, labeled as A–I (Figure 8). Sediments extracted from the north section of Trench 1 at JKF-1 (Figure 9), comprise an interstratified sequence of sands and silts, reflecting changing environmental conditions. The sequence is underlain by well to moderately-well sorted medium sands with a near symmetrical skewness, representing aeolian transport under arid environmental conditions. This is overlain by Unit H - the exclusive artefact-bearing stratum, which is a consolidated, pale yellowish-grey silty sand mottled with orange streaks. An increase in magnetic values likely reflects the presence of artefacts, while statistical analysis of the grain size characteristics reveal the unit to be poorly sorted with a minor silt-sized component, possibly indicative of reworking by water. The unit also features abundant rhizoliths (indicative of increased former vegetation) along with occasional modern roots and rootlets. Subsurface animal burrows were observed in the trench, including a large burrow running down the centre of the trench that contained fox bones.

The artefact-bearing unit is overlain by Units G and E, which display a fining up sequence from cemented silt-sand sediments, into lacustrine material. Increases in magnetic values throughout the units correspond to grain size changes, while organic content (LOI_550_) also increases, achieving substantially high values at ca. 30 cm. Similarly, carbonate content (LOI_950_) values are increased throughout Unit E, reflecting the precipitation of lake carbonate material. Unit D reflects an influx of aeolian sand and a return to arid conditions, with low organic content and carbonate values. This is overlain by Units C4 and C1, which represent the formation of calcrete and reflects a substantial increase in humidity within the region. Analysis of the grain size characteristics from these calcrete units reveal the sediments to be very poorly sorted silts with a near symmetrical skewness. These are overlain by aeolian sediment (Unit B), which reflects the onset of arid conditions within the region.

All excavated sediment was sieved through a 6 mm^2^ mesh. A total of 300 artefacts were recovered from Unit H, most of which were small (<2 cm). A total of 30 larger artefacts were piece-plotted in place. Unit H corresponds with the point at which artefacts are concentrated on the sloping mound surface, indicating that the surface artefacts derive from the deposit, as confirmed by refits.

We used OSL dating to obtain burial ages for sand-sized grains of quartz. Three samples were taken, one from Stratum I (JKF-OSL-4), the lowermost excavated level, and two from Stratum H (JKF-OSL1, JKF-OSL-2), the artefact-bearing level. In each of the three samples, only between 1–2% of the grains were suitable for equivalent dose (D_e_) determination. The single-grain D_e_ values were widely spread, with overdispersion values ranging between 35±5% and 62±8% (see Supporting Information S2), most likely indicating intrusion of younger grains into the host sediments. The finite mixture model [31], [32] was used to estimate the minimum number of discrete populations of grains needed to fit these D_e_ distributions, and two populations of grains accounted for 90–95% of the total number of D_e_ values. For the lowermost sample (JKF1-OSL1), 74% of the D_e_ values yielded a depositional age of 64±6 ka, with a smaller population of grains deposited 26±5 ka ago. The artefact-bearing level of Unit H had depositional ages of 87±6 ka, with a population of younger grains at 49±5 ka (JKF1-OSL4); and 86±11 ka, with a population of younger grains that were last exposed to sunlight 53±6 ka ago (JKF1-OSL3). The two main Unit H populations correspond, therefore, to burial ages of ∼90–85 ka (MIS 5a–b) and ∼50 ka (MIS 3). As each of these populations contains an approximately equal number of grains, it is difficult to ascertain the period of human occupation, although on the basis of overlying strata, it is conceivable that the younger MIS 3 grains were intrusive into an MIS 5a–b deposit. Regardless of precise age, the OSL estimates indicate that occupation most likely occurred during a humid interval.

The 1222 artefacts from Unit H and the surface are technologically homogeneous and are treated here as a single assemblage. The assemblage consists of 1113 pieces of debitage (91%), 99 cores (8%) and 10 lightly retouched tools (1%) (Table 1; Figures 10–11). Ferruginous quartzite dominates the assemblage (76%), followed by quartz (22%), and rarely, rhyolite (1%) and other materials (1%) (Table 2).

The cores made from quartz (n = 61) and quartzite (n = 37) vary typologically, likely owing to raw material type and clast size (Table 3). Single platform cores from quartz are the most common form and high percentages of simply flaked amorphous cores and fragments in quartz and quartzite were also present. A total of 39 cores, produced from both quartzite and quartz, could be classified as Levallois, representing 34% of the entire core assemblage (Table 3). The most common type of Levallois core was preferential Levallois with centripetal preparation and unidirectional convergent preparation, accompanied by other cores that could be classed as non-preferential recurrent centripetal Levallois. Non-preferential bidirectional and recurrent unidirectional techniques were more rarely used. Two quartzite cores show use of the unidirectional convergent Levallois method, indicating the production of triangular-shaped flakes and Levallois points. Radial, discoidal and bidirectional cores accompanied the Levallois cores. Blocks of quartzite, which were locally available, were not necessarily always used as directly modified cores, as Levallois debitage was also manufactured on large quartzite flakes. Artefact refits have been identified, including a set of refitting quartzite flakes and two refitting rhyolite flakes. One of the rhyolite flakes was found in excavation and another on the surface, supporting the proposition that the surface material was derived from Stratum H.

The majority of the debitage are flakes (n = 744), with the recovery of only small numbers of blades (Table 4). Faceting occurs on 24% of the flake blanks. Considering the relatively large size of the assemblage, only a small number (n = 11) of lightly retouched tools was recovered (Table 5, Figure 10). Retouch is not invasive and mostly confined to lateral margins. Although not strongly represented in the cores, likely owing to subsequent reshaping, the blanks show that unidirectional-convergent Levallois reduction, producing Levallois points and triangular flakes, was important in the JKF-1 assemblage (Figure 10). Hard hammer percussion appears to have been exclusively used. The platforms and scar patterns observed on blanks indicate maintenance for the exploitation of one single debitage surface (the Levallois surface) on the cores.

Given the relatively good preservation conditions at JKF-1, a feasibility study of artefact residues was conducted (Supporting Information S3). The seven selected artefacts had generally fresh edges and included 6 quartzite and 1 quartz artefact. The selected types and forms (2 Levallois points, 2 preferential Levallois flakes, 1 Levallois flake, 1 blade, 1 flake) were considered to be potentially good candidates for use. Visual inspection and low powered microscopy was first employed to screen the artefacts for possible residues. Screening was followed by the removal of residues from particular edges of artefacts. The removed residues were subsequently assessed using absorbance spectroscopy and gas chromatography coupled mass spectrometry (GC/MS) at Lakehead University (Canada). Five of the seven artefacts produced positive results for compounds consistent with utilization of plants and animals (Table 6, Supporting Information S3). Two artefacts had plant residues (nos. 847, S-20; Figure 11), one had animal residue (no. 873, Figure 11), and two had a combination of both animal and plant (nos. 504, S-76; Figure 10). The identification of combined plant and animal residue on the two Levallois points (nos. S-76, 504) may be consistent with hafting and meat acquisition and use. The identification of plant residues on the preferential Levallois flakes (nos. S-20, 873) and the Levallois flake (no. 847) may be a sign of the use of carefully prepared items. The identification of plant and animal residues on plain edges (nos. 504, 873) and on edges that showed limited signs of technological damage (no. 847) and minor nibbling (no. 521, Figure 10) indicates that items other than tools may have been used for particular and brief tasks. It is notable that a plain faceted flake (no. 521) and a plain blade (no. 209, Figure 11) did not yield authentic residues. The results of this feasibility study are encouraging, and indicate that residues can survive in arid environments over a long period of time.

Most of the artefacts from JKF-1 are ferruginous quartzite, originating from an outcrop (JKF-12) (27°55′15.1 N, 40°48′05.9 E) at the base of the jebel which is ∼800 m to the west. At JKF-12, the quartzite occurs as spatially extensive beds of relatively fine-grained material available as large clasts. The quartzite was reduced at the base of the jebel, which served as a location for raw material procurement based on the identification of large Levallois cores measuring up to 25×20 cm (thereby contrasting with the typically much smaller cores at JKF-1). Within the sandstone are naturally occurring quartz cobbles and pebbles, which are also widely distributed across the basin floor. The quartz occurs within a few hundred meters of the site, while site-to-source distance for the transport of ferruginous quartzite is no greater than 800 m. The rare rhyolite artefacts suggest long distance transport.

In sum, we interpret the JKF-1 site to be a relatively short-term occupation given the presence of lithic assemblages from a single and thin deposit, the homogeneity of the lithic assemblage, and the presence of refitted cores and flakes. Based upon mapping and stratigraphic analyses, occupations appear to be on an aeolian dune, overlooking a low-lying area that may have had standing water (Figure 12).

### Jebel Umm Sanman

Jebel Umm Sanman is the largest jebel in the area, measuring 7 km north-south and 3 km east-west, and reaching a height of 1264 m. Jebel Umm Sanman 1 (JSM-1) (27°58′34.83 N, 40°55′28.28 E) is located in the southeastern margin of the jebel, overlooking the western margin of the northern palaeolake (Figure 1, 13). The site occurs near a single, narrow seam of distinctive yellow fine-grained quartzite at the base of the sandstone bedrock, at a height of ca. 820 m (Figure 1, 13).

Low densities of artefacts were found over a surface measuring ca. 160×75 m, and across a slight slope going west to east (Figure 14). Two one meter square units were placed in a flatter area and in the central portion of the site to ascertain whether buried artefacts could be recovered. Upon removal of a 5–10 cm aeolian sand and recent colluvium, a 50–60 cm thick deposit with artefacts throughout was uncovered. The deposit was divisible into two strata, a pink-grey (Stratum B) and pale greyish yellow (Stratum C) sand with abundant weathered sandstone pieces resting on sandstone bedrock. Artefacts were found throughout Strata B and C.

Two OSL samples were taken from the artefact-bearing levels, one from Stratum B (JSM1-OSL1), 20 cm below surface, and one from Stratum C (JSM1-OSL2), 42 cm below surface. In each of the two samples, only ∼1% of the grains were suitable for D_e_ determination. As at JKF-1, the single-grain D_e_ values were widely spread, with overdispersion values of 93±11% and 67±10% (see Supporting Information S2); these high values most likely reflect the shallow nature of the excavation and the intrusion of younger grains into the host sediments. The finite mixture model [31], [32] was used to fit these D_e_ distributions and, in both cases, two populations of grains were sufficient to account for more than three-quarters of the total number of D_e_ values. For the Stratum C sample (JSM1-OSL2), the two D_e_ populations yielded burial ages of 140±14 ka and 61±8 ka, while in Stratum B (JSM1-OSL1) the ages were 96±9 ka and 42±9 ka (the former age for the Stratum B sample being associated with ∼60% of the grains). Given the amount of post-depositional mixing of grains evident in these two samples, it is not clear which population of grains is most appropriate to determine the age of the artefacts, but the time of burial can conservatively be constrained to between ∼140 ka and ∼40 ka, with the most likely interval being 100–60 ka, or between MIS 5c and MIS 4. Human occupation probably occurred during a humid period in MIS 5, with MIS 4 sand grains contributing to artefact burial.

The 11 surface and 77 buried artefacts were technologically homogeneous. The 88 artefacts included 74 pieces of debitage (84%), 10 cores (11%) and 4 retouched pieces (5%) (Table 1; Figure 15). The majority of the artefacts were made from the local yellow quartzite (92%), with quartz (5%) and other materials (3%) making up the remainder (Table 2). With the exception of two fragments, the cores are all exclusively Levallois, with 2 classifiable as Levallois preforms and 5 as preferential Levallois with centripetal preparation. The majority of the debitage consists of flakes, with 14% showing faceted platforms. Only one retouched piece was recovered, a side retouched flake. One retouched point and two bifacial pieces are present (Figure 15).

## Discussion

We have discovered and investigated three stratified and buried lithic assemblages in the Nefud Desert, thereby nearly doubling the count of well preserved Middle Palaeolithic sites in the Arabian Peninsula. The geographic location of the Jubbah sites, in the northern interior region of Arabia, adds important new information about the distribution of Middle Palaeolithic hominins, as the other published site excavations are located in the extreme southern parts of the peninsula. Out of Africa dispersal models have mostly emphasized the Bab al Mandab route on the basis of genetic data [33], [34], [35] and archaeological discoveries [19], [20], [36]. Yet, other researchers have pointed out that alternate dispersal routes from Africa to Arabia were possible during humid periods [27], [37], [38]. Indeed, major riverine routes made it possible for hominins to enter into the peninsula from several directions, particularly from the Levant and the Sinai, the Mesopotamian plains and Euphrates riverine zone, and the Persian Gulf (Figure 16a, Supporting Information S1). Travel across Arabia, either from the north or from the south, was possible owing to a dense network of major rivers and tributaries. The presence of a dense network of river channels in the region surrounding the Nefud Desert implies that hominins could have reached the Jubbah palaeolake using different routes, including from: 1) the north and west, travelling up the tributaries and headwaters of the Wadi as Sirhan; 2) the north and northeast, from the riverine plains of Mesopotamia and the Euphrates basin; and, 3) the Persian Gulf in the east, moving up to the headwaters of the Wadi al Batin (Figure 16). Mapping of the Jubbah palaeolake reveals it was not a unique or isolated phenomenon, as it was part of a network of other palaeolake basins (Figure 16b) that have not yet been explored for archaeological sites. The Jubbah palaeolake must be viewed as a component of a significant river and stream distribution network in northern Arabia, implying many possibilities for hominin movements across the Nefud Desert and the surrounding region.

The environmental record from JQ-1 and JKF-1 reveals the presence of a series of palaeosol, calcrete and lacustrine deposits that formed under a substantially more humid and ameliorated climate. Multiproxy, sedimentological and stratigraphic evidence indicates that lakes within the Jubbah and Katefeh basins fluctuated in size, in response to changing climatic conditions between MIS 7 and MIS 4. During peak pluvials such as MIS 5e, lakes within these basins may have covered an area of up to 78 km^2^. Although lake volumes may have been reduced during less humid conditions, soil formation occurred along lake margins while the surrounding landscape remained dominated by grassland, with some trees. This is an ideal setting for migrating animals, which in turn, would have been attractive to mobile hunter-gatherers. We note that this humid period migratory scenario contrasts with genetic data suggesting dispersals at the outset of an arid phase, or MIS 4 (between 70–60 ka) [35], [39].

Having reached Jubbah, Middle Palaeolithic hominins would have resided on the margins of the palaeolake, which would have provided an attractive habitat with key resources. Sites were situated on the western and southern parts of the northern lake (JSM-1, JQ-1) and on the eastern fringes of the southern lake (JKF-1). In the case of JKF-1, geomorphological reconstruction reveals that the occupation occurred on an aeolian sand dune, overlooking the lower-lying lake (Figure 12). The site environment was a grassland with some trees probably fringing the lake and the rivers that flowed into the lake basin. The sites were located at the base of the Umm Sanman Jebel and in close proximity to Jebel Qattar (100 m) and Jebel Katefeh (800 m), where a plentiful supply of raw material was accessed in each case. The ubiquity of raw materials resulted in low retouch intensity, as conservation of materials was not necessary, and the expedient use of plain and un-retouched flakes, as demonstrated by residues on their edges. The flakes and tools appear to have been used in the processing of plants and animals, as evidenced at JKF-1. Middle Palaeolithic hominins appear to have ranged over a wide area, incorporating the Jubbah basin complex, as exemplified by the 24 km distance between JQ-1 and JKF-1. The use of rhyolite and flints may imply that the Jubbah hominins were probably procuring material from further distances, as local exposures of these materials have not been identified.

Though so far a small excavated stone tool assemblage, the recovery of 28 artefacts in a deposit dated to 211±16 ka represents the oldest reliably dated occurrence in the Arabian Peninsula. We tentatively associate this assemblage with the Middle Palaeolithic on the basis of the age of the technology and the recovery of two Levallois flakes. Although we cannot be certain of the species that manufactured the artefacts, we note that the lithic assemblages were produced at a time corresponding with the origin of *Homo sapiens* in Africa based on mitochondrial DNA [40] and nuclear genomic [41] age estimates and fossil finds [42], [43]. The early JQ-1 artefacts also correspond with the upper age range limits of the Acheulo-Yabrudian and the Zuttiyeh fossil, potentially indicating the presence of archaic hominins [44] in Arabia, and possibly early representatives of the Neanderthals [45].

An entirely different scenario presents itself 130,000 years later, as the date of 75±5 at JQ-1 generally corresponds with the occupation of the Levant by *Homo sapiens* and the Neanderthals. The MIS 5a age result at JQ-1, and the MIS 5 ages at JKF-1 and JSM-1, overlap with mitochondrial DNA coalescence age estimates for the dispersal of *Homo sapiens* between 75 and 62 ka [46] or earlier [34], [47]. The wet period of MIS 5 has been considered the environmental setting for the movement of Middle Palaeolithic hominins across southern Asia, possibly representing a range expansion eastwards of modern humans before the Toba super-eruption of 74 ka [48], [49], [50]. This hypothesis has been disputed on the basis of the somewhat younger age range estimate of the L3 haplogroup at ∼70–60 ka [39] and the argument that the expansion of *Homo sapiens* is marked by crescentic tool technologies and other modern human behavioural traits [6], [51]. Yet, recent support for an MIS 5 expansion of *Homo sapiens* comes from archaeological finds of characteristic Middle Palaeolithic technologies in Arabia in MIS 5e–c [19]–[20] and nuclear genomic estimates which indicate that the split between Africans and non-Africans occurred as early as 130 to 90 ka [41], consistent with fossil finds of *Homo sapiens* in the Levant [52], [53] and at the time of possible interbreeding of *Homo sapiens* and Neanderthals [54]. These controversies indicate the need to recover hominin fossils in Arabia, which is feasible given the identification of Pleistocene mammalian fauna in a nearby lake basin of the Nefud [24], [55].

The Jubbah sites demonstrate stone tool reduction methods that are characteristic of Middle Palaeolithic industries. The lithic assemblages contain a variety of reduction methods, including centripetal, bidirectional, unidirectional and convergent techniques. The JQ-1 and JSM-1 assemblages are primarily centripetal in character, with centripetal preparation of preferential Levallois cores at the latter. At JKF-1, it appears that the earlier phase of reduction of quartzite was primarily unidirectional and unidirectional convergent, with a move towards centripetal reduction as size decreased. At JKF-1, quartz was usually reduced in a simple flaking manner, but some examples are rather more sophisticated and are Levallois-like. The method of manufacture of the Jubbah industries differs from the more distinctive techniques described in the extreme southern zones of Arabia, including the Nubian technocomplex in Oman [20] and the production of flakes and blades from flat debitage surfaces at SD-1 in Yemen [22]. Rose et al. [20] have argued that the presence of Nubian core methods in Oman are directly tied to the presence of *Homo sapiens* in Arabia in MIS 5, whereas Delagnes et al. [22] have argued that the MIS 3 blade and point dominated assemblages in Yemen are broadly reminiscent of techniques that Neanderthals were using in the Levant. The unidirectional convergent technique practiced at JKF-1 and the presence of Levallois points shares some similarities with assemblages in the Levant. Yet, the recovery of a retouched point at JQ-1 and two bifacial pieces at JSM-1 tentatively suggest an affinity with the African Middle Stone Age as opposed to the Levant, where such tool types are absent. Distinctive foliate bifaces, which are present in Africa and southern Arabia, are so far unknown at Jubbah and in northern Arabia. The newly emerging information implies that distinctive technological methods were being used in different geographic areas of Arabia in MIS 5 and in MIS 3. Stylistic tool assemblage variation indicates different traditions in stone tool-making, thus suggesting different adaptations and possibly different source populations.

To situate the Jubbah technology in a wider geographic context, 55 cores from JQ-1 (upper assemblage) and JKF-1 were compared to those from neighbouring regions. The comparative sample included Middle Stone Age sites from the Horn of Africa; the Levantine Mousterian sites of Tabun Cave, Layer C, and El Wad; the early *Homo sapiens* site of Skhul in the Levant; Aterian sites in North Africa; the Middle Palaeolithic levels of the Haua Fteah in Libya; and Middle Palaeolithic sites from both above and below the Toba ash in India. To assess the relative similarity of core technologies, a Discriminant Functions Analysis was performed (Table7, Supporting Information S4). Figure 17 shows the group centroids for the different core groups. The comparative analysis indicates that the Jubbah cores most closely correspond with each other and Middle Palaeolithic assemblages from Tabun Layer C, which has produced human remains that have variously and controversially been either classified as Neanderthals or as *Homo sapiens* [56], [57]. The Aterian cores are similar to those from the Haua Fteah and to a lesser extent Skhul, which perhaps reflects their geographic relations and their association with *Homo sapiens*. The Indian cores have previously been shown to be similar to sub-Saharan Middle Stone Age industries [49], [58], and yet they differ from those found at Jubbah. In general, the Jubbah cores are not akin to the early North African and Levantine assemblages produced by *Homo sapiens*, perhaps signaling a complicated demographic history, and indicating limitations of using particular stone artifact types as proxies for hominin species and populations [59], [60].

In conclusion, this article presents the first systematic research on archaeological sites in the Nefud Desert of Saudi Arabia. Our analysis indicates that Middle Palaeolithic hominins were able to penetrate deep into the interior of northern Arabia during ameliorated humid periods. Middle Palaeolithic archaeological evidence for terrestrial and inland movements contrasts with, or at least complicates, the coastal model for rapid human expansion [33], [61] by groups using cresentic and blade technologies [6]. It remains unclear what hominin species was responsible for the manufacture of the earlier and later stone tool assemblages from Jubbah, and it is entirely possible that more than one species was involved. Our palaeoenvironmental analyses indicate that hominins were able to utilize and adapt to the marginal environment of the Nefud during humid periods. It remains unclear what happened to these hominins during the arid and hyper-arid periods of the Late Pleistocene. Small populations of Middle Palaeolithic hominins may have contracted and survived in refugium zones in Arabia [19], [22], [62], [63], although genetic interchanges and local extinctions are expected [41], [60]. In reality, on-the-ground investigations are beginning to show that wet and dry phases may show north-to-south timing differences across the Levant and the Syro-Arabian Desert, as well as latitudinal variations of wet conditions between basins, implying more complicated demographic processes of expansion, contraction and extinction [64]. Though emerging information indicates that Arabia is rich in its Palaeolithic archaeological record, it appears as though population size was not as large and dense compared to other more attractive habitats and regions [65], [66], [67]. Though further interdisciplinary research programmes and archaeological investigations are required, current research in the Nefud Desert and in other parts of Arabia are beginning to fill in major gaps in our understanding of the demographic and evolutionary history of hominins. This frontier research needs to be furthered by conducting surveys and excavations in various environmental zones of Arabia in order to recover ecological data and information on hominin population history and changing adaptive behaviours.

## Materials and Methods

### Ethics

All necessary permits for the Jubbah fieldwork and analyses were obtained from the Saudi Commission for Tourism and Antiquities, Kingdom of Saudi Arabia.

## Supporting Information

Supporting Information S1 Mapping and palaeohydrological methods report.
(DOCX)

Supporting Information S2 Supporting information for OSL dating.
(DOC)

Supporting Information S3 Residue analysis report.
(PDF)

Supporting Information S4 Core analysis report.
(DOCX)
