# Supplementary material for: Hominin Dispersal into the Nefud Desert and Middle Palaeolithic Settlement along the Jubbah Palaeolake, Northern Arabia
Source: PLoS One. 2012 Nov 19;7(11):e49840. doi: 10.1371/journal.pone.0049840 (PMC3501467; doi:10.1371/journal.pone.0049840)
Supplement: Supporting Information S1 — Mapping and palaeohydrological methods report. (DOCX) [file pone.0049840.s001.docx]

## Supporting Information S1: Mapping and Palaeohydrological Methods Report

**Hominin Dispersal into the Nefud Desert and Middle Palaeolithic Settlement along the Jubbah Palaeolake, Northern Arabia**

**Michael D. Petraglia, Abdullah Alsharekh, Paul Breeze, Chris Clarkson, Rémy Crassard, Nick A. Drake, Huw S. Groucutt, Richard Jennings, Adrian G. Parker, Ash Parton, Richard G. Roberts, Ceri Shipton, Carney Matheson, Abdulaziz al-Omari, Margaret-Ashley Veall**

In order to define palaeodrainage systems for the Arabian peninsula, GIS-based D8 flow accumulation analyses [1] were applied to the Hydrosheds 3 arc-second (c. 90m) resolution flow direction dataset [2],[3]. A threshold was then applied to represent a stream by selecting a minimum required contributing area for cells to be defined as a stream. Two thresholds were applied in order to generate two stream networks at different spatial scales. The first defining the detailed palaeodrainage in the Nefud region, in the vicinity of the JQ-1 site (Figure 16B), and the second defining the major drainage systems at a peninsula-wide scale (Figure 16A).

To map channel networks in the Nefud region a stream delineation threshold of 12345 cells (providing a minimum contributing catchment area of c.100km^2^ at the equator) was employed in order to define a detailed network. This threshold was shown by examination of remotely sensed data to be an underestimate of true former drainage network density in most areas; however, thresholds below this level were found to provide stream networks that were so complex as to inhibit effective display of the data in Figure 16B, and may represent stream headwaters of more ephemeral character.

Palaeolakes were also mapped for the Nefud region in order to provide a comprehensive depiction of the palaeohydrology. We used the Hydrosheds DEM [2] for this because it lends itself to mapping desert palaeolakes as it identifies and preserves endorheic (closed) basins, which are regions where palaeolakes are found. In humid areas rivers that flow into closed basins fill them up until they overspill into a channel, forming a lake and associated outflow channel that eventually drains into the sea. Channel network mapping algorithms were designed for humid regions where closed basins are full but when implementing them problems were found when the accumulation frequently terminated in pits in the DEM caused by noise and errors. To overcome the problem of ‘broken networks’ pits are routinely filled before flow accumulation is applied, thus providing continuous channel networks. However, in deserts filling all the depressions results in the removal of all closed basins and thus masks the presence of palaeolakes that might have existed in past humid periods within the closed basins that have been filled. Hydrosheds recognises this problem and only the small pits, that are generally errors in the DEM, have been filled. The larger closed basins have been preserved. Applying flow accumulation thresholds to Hydrosheds produces channel networks that drain into these basins where appropriate, indicating the locations of the basins, and thus palaeolakes.

Investigation of the Hydrosheds network showed that in many areas river channels terminated in large closed basins that could have contained palaeolakes. However, this was not always the case, in other places a drainage network that appeared to have formerly belonged to a single connected system was mapped as many separate channel segments. Investigation of the morphology of these areas usually indicated that they had been truncated by aeolian dunes that were blocking the channel. To determine whether palaeolakes or dunes caused each broken channel network segment, further investigations were performed using Landsat TM and ETM+ data with a spatial resolution of 30 meters and the SRTM version 4 DEM with a 90 meter resolution [4]. To map the presence of palaeolake deposits Landsat false colour composites (FCC) were generated using a band 7,4,1 red, green and blue combination that was found to be effective for this purpose in the Sahara (5, 8), delineating the lakes in a light blue colour. The effectiveness of this method in the Nefud was confirmed by the fortuitous capture of a Landsat TM image soon after an exceptional rainfall event that temporarily partially filled the palaeolakes (Figure S1). This event confirmed our interpretation of palaeolakes by showing that these light blue features became much darker after rainfall due to the presence of water, whilst the rest of the landscape remained similar, apart from the growth of vegetation in river channels and around lake margins that briefly received substantial amounts of floodwater.

If palaeolake deposits were identified in a closed basin at the termination of a channel, as is the case in Figure S2, then the system was interpreted as having fed a palaeolake, the area of which was mapped by digitizing the extent of the palaeolake deposit. If no palaeolake was present and the channel appeared to continue further downslope after the break, it was likely that the network was severed by Aeolian activity and the Landsat ETM+ imagery and DEM were investigated for the presence of dunes. If dunes were found, but palaeolake sediments were also found within the channel on the upstream side of the dune, then it was concluded that the dune blocked the channel during, or prior to, the last wet phase, when a lake formed against the blocking dune. In these circumstances, the palaeolake was mapped and the network remained terminated. If no palaeolake sediments were evident then it was assumed that the channel blockage had occurred since the last wet phase and the DEM data was used to determine the likely former channel path, which was then digitised, connecting the once severed channel segments. After implementation of these procedures some channels still exhibited breaks even though they were connected by continuous river terraces on each side of the channel and were only separated by topographic highs of a few meters in altitude. This situation indicates past connections but provides no evidence as to when or how any blockage occurred. We connected these networks on the assumption that they must have been flowing in wet phases in the not too distant past. Implementation of these methods produced the likely major drainage networks active during recent wet phases on the Arabian Peninsula, those since dune activity started to block channel networks.

A less detailed palaeodrainage map was developed of the main Pan-Arabian drainage systems by applying a coarse threshold of 123456 cells (equating to a minimum catchment area of c.1000km^2^ at the equator) to the Hydrosheds data to define solely the larger drainage features of the peninsula (Figure 16A). The modelled network was then manually thinned for display clarity to define only the principal connected systems that may have provided potential dispersal routes for hominins. In the sand seas the channel networks were often particularly dense and here the small-scale network was further pruned through classification of the stream network according to the Strahler stream order system [5], and removal of all first order streams intersecting areas of Quaternary aeolian deposits defined by the USGS Geo2BG geological data [6]. This process reduced the network to only the largest drainage routes. Channel network reconnection was then achieved using the same workflow as the more detailed Nefud palaeohydrology outlined above, though in this case palaeolakes were not mapped because they are generally too small to display at this scale.

With the exception of the Rub al Khali and Nefud sand seas, river networks in both Figure 16A and B were found to agree with channel networks indicated in the DEM and remotely sensed data to an excellent level. In areas examined in detail using remotely sensed and DEM datasets the method was found to have successfully defined palaeochannels even where enhanced processing was required to elucidate these features in these data. The density of former channels implied by these observations is impressive, particularly when it is considered that the utilised thresholds appear to be an underestimate of the total former networks, and will not identify any relict systems represented by inverted topography, or completely in filled systems located on planar surfaces.

In the major sand seas of the Rub al Khali and Nefud, dense channel networks were produced between successive lines of linear dunes, but channels could not be seen in either the DEM or the Landsat imagery, presumably because they were since buried under active sand. Though there is no direct evidence of palaeochannels, in the case of the Nefud, flow pathways in the dune areas can be seen connecting to deeply incised palaeochannels outside the dune area, and to palaeolakes in interdune depressions within the sand sea, as at Jubba; thus implying either the successful definition of underlying flow directions in the interdune areas, or long-term dune stability in the Nefud region, or both. Given the likelihood that the plotted flow paths within the Nefud sand sea accurately reflect some characteristics of prior flow, these networks have been included in the detailed palaeodrainage network (Figure 16B), however, their exact location and density should be treated with caution because dune mobility could have changed their morphology and location during intervening arid phases. Sedimentological evidence shows that dune mobility though clearly present is probably limited, with some interdune depressions showing remnants of past MIS 5 and 7 palaeolakes, suggesting past erosion and sand activity [7] but with others showing evidence for long term stability with thick sequences of palaeosols in MIS 5 dune sands preserved under a 30 m thick layer of more recent active sand [8].

**References**

1. Jenson SK, Domingue JO (1988) Extracting topographic structure from digital elevation data for geographic information system analysis. Photogrammetric Engineering and Remote Sensing 54:1593-1600.

2. Lehner B, Verdin K, Jarvis A (2008) HydroSHEDS Technical Documentation (v 1.0). 1-27. Available at: http://gisdata.usgs.gov/webappcontent/HydroSHEDS/downloads/HydroSHEDS_TechDoc_v10.pdf [Accessed April 19, 2012].

3. Lehner B, Verdin K, Jarvis A (2008) New global hydrography derived from spaceborne elevation data. EOS, Transactions of the American Geophysical Union 89:93-94.

4. Jarvis A, Reuter H., Nelson A, Guevara E (2008) Hole-filled seamless SRTM data V4, International Centre for Tropical Agriculture (CIAT). Available at: http://srtm.csi.cgiar.org.

5. Strahler A (1957) Quantitative analysis of watershed geomorphology. Transactions of the American Geophysical Union 38:913-920.

6. Pollastro RM, Karshbaum AS, Viger RJ (1999) Map Showing Geology, Oil and Gas Fields and Geologic Provinces of the Arabian Peninsula. U.S. Geological Survey Open-File Report OFR-97-470-B (Denver, Colorado.).

7. Rosenberg TM (2011) Palaeoclimate history of the Arabian Peninsula : humid phases recorded in lake deposits. Unpublished PhD thesis. University of Bern.

8. Petraglia MD, Alsharekh AM, Crassard R, Drake NA, Groucutt H, et al. (2011) Middle Paleolithic occupation on a Marine Isotope Stage 5 lakeshore in the Nefud Desert, Saudi Arabia. Quat Sci Rev 30: 1555–1559.


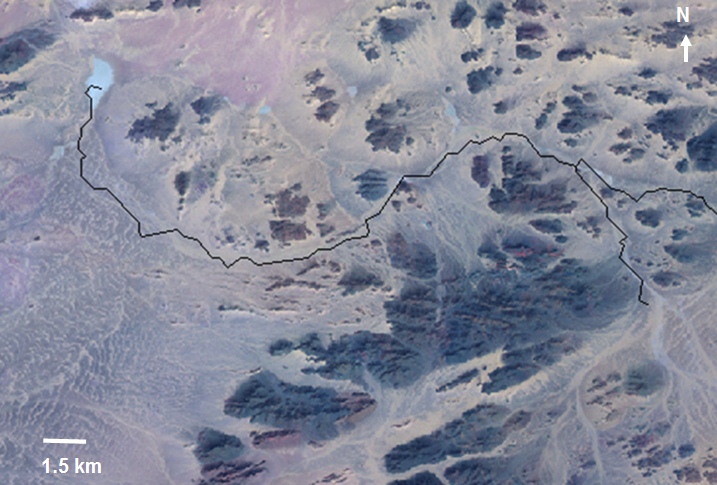


**Figure S1. Example Landsat TM FCC image (7,4,2 RGB band combination) produced to highlight palaeolake deposits (light blue).** Image is overlain by the detailed drainage network (black). Note termination of drainage path in an endorheic depression holding palaeolake deposits.


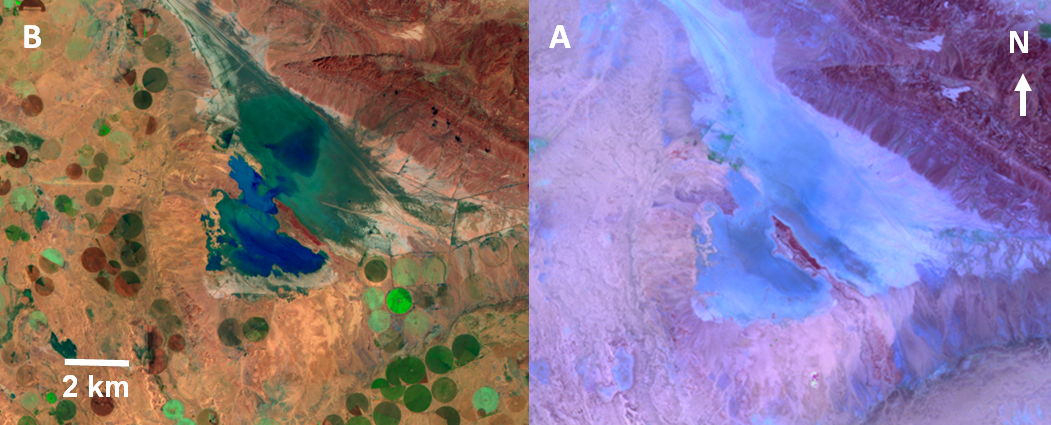


**Figure S2. Palaeolake deposits from the Nefud region.** A Landsat TM FCC from the 19^th^ of May 1985 showing palaeolake deposit extent (light blue). B Landsat TM FCC image of the same area from the 16^th^ of January 1990, with accumulation of water visible within the basin, following an exceptional rainfall event. Note also the expansion of pivot irrigation agriculture (the circular features) during this time.
