# Supplementary material for: Hominin Dispersal into the Nefud Desert and Middle Palaeolithic Settlement along the Jubbah Palaeolake, Northern Arabia
Source: PLoS One. 2012 Nov 19;7(11):e49840. doi: 10.1371/journal.pone.0049840 (PMC3501467; doi:10.1371/journal.pone.0049840)
Supplement: Supporting Information S2 — Supporting information for OSL dating. (DOC) [file pone.0049840.s002.doc]

## Supporting Information S2: OSL Dating

**Hominin Dispersal into the Nefud Desert and Middle Palaeolithic Settlement along the Jubbah Palaeolake, Northern Arabia**

**Michael D. Petraglia, Abdullah Alsharekh, Paul Breeze, Chris Clarkson, Rémy Crassard, Nick A. Drake, Huw S. Groucutt, Richard Jennings, Adrian G. Parker, Ash Parton, Richard G. Roberts, Ceri Shipton, Carney Matheson, Abdulaziz al-Omari, Margaret-Ashley Veall**

Optically stimulated luminescence (OSL) dating of individual sand-sized grains of quartz from the Jebel Katefeh (JKF1) and Jebel Umm Sanman (JSM1) samples was performed using the same equipment and data analysis procedures as those described in detail previously (Jacobs et al., 2008, 2011, 2012, in press), so only a brief account is provided below. Readers are referred to Jacobs and Roberts (2007) for a review of single-grain OSL dating in archaeological contexts, and to Galbraith and Roberts (2012) for a recent overview of the statistical models and graphical displays employed here and in OSL dating more generally.

Samples JKF1-OSL1 and JKF1-OSL4 were collected from depths of 106 cm and 80 cm, respectively, in the north face of the excavation trench at Jebel Katefeh. JKF1-OSL4 was taken from Stratum H—the layer containing the artefacts—whereas JKF1-OSL1 represents a sample of the underlying, culturally sterile sands. JKF1-OSL3 was also collected from Stratum H, but from a depth of 42 cm in the south face of the trench, ~3 m west of JKF1-OSL1 and ~2.5 m west of JKF1-OSL4, where the artefacts were more concentrated. At Jebel Umm Sanman, samples JSM1-OSL1 and JSM1-OSL2 were collected from depths of 20 cm and 42 cm, respectively; artefacts were recovered from both of these levels.

OSL samples were collected by hammering opaque plastic tubes into the cleaned section faces, and then sealing the tubes in black plastic bags after removal. An estimate of the *in situ* gamma dose rate was made in the field, by inserting a NaI(Tl) detector into the holes created by removing these tubes. Additional sediment samples were collected for laboratory measurements of field water content and beta dose rate, the latter by means of low-level beta counting and high-resolution gamma spectrometry (HRGS). Under safe-light conditions, quartz grains of 180–212 μm in diameter were extracted from the internal portions of the tubes, and prepared for OSL dating using the same procedures as those described by Jacobs et al. (2008, 2011, 2012).

Measurements were made on between 2500 and 4000 individual grains of each sample (see Table S2.1), and the single-aliquot regenerative dose (SAR) procedure was used to estimate the equivalent dose (D_e_) values for grains that passed the same set of reliability criteria as those used by Jacobs et al. (2008, 2011, 2012); these criteria are explained in the footnotes to Table S2.1. Each grain was optically stimulated by a green laser beam for 2 s at 125°C and the resulting ultraviolet OSL emissions detected by an Electron Tubes Ltd. 9235QA photomultiplier tube fitted with Hoya U-340 filters. D_e_ values were estimated from the initial 0.2 s of OSL decay, and the mean count rate over the final 0.3 s was subtracted as background. Before laser stimulation, grains were preheated at 260°C for 10 s (natural and regenerative doses) or at 220°C for 10 s (test doses), and possible contamination of the quartz grains by feldspar inclusions was checked using infrared stimulation.

These measurement conditions and signal integration intervals were chosen on the basis of dose recovery tests (Galbraith et al., 1999) performed on 1900 individual grains of quartz from sample JKF1-OSL4, which we exposed to natural sunlight and then gave a beta dose of 52 Gy in the laboratory. Of these grains, only 42 (2.2% of the total number measured) satisfied all of the reliability criteria and 86% of these (*n* = 36) gave measured doses within 2σ of the correct (known) dose; this proportion is only slightly less than the statistical expectation of 95%. The additional spread in the recovered doses (termed ‘overdispersion’) is 9 ± 4%, and the mean ratio of measured to given dose is 0.89 ± 0.03 (*n* = 42). The latter value is not statistically consistent with the desired value of unity (and this was also the case for the other dose recovery conditions tested), but we note that only 4 of the 42 recovered doses were measured with relative precisions of better than 10% (see Fig. S2.1d). If this fractional shortfall in measured dose applies to the natural samples, then the D_e_ values and OSL ages listed in Table S2.2 may be slightly underestimated.

The number of grains measured, rejected and accepted for each sample, and the reasons for their rejection, are listed in Table S2.1. The vast majority of measured grains (88–92%) were rejected because their test dose OSL signals were indistinguishable from background, resulting in D_e_ values being obtained from only 1–2% of the total number of measured grains. These grains, like those from the nearby site of Jebel Qattar (JQ1)—see Fig. S1a in Petraglia et al. (2011)—were characterised by a rapid rate of initial OSL decay when stimulated by the laser beam, consistent with the OSL signal arising from the most light-sensitive (‘fast’) component of quartz OSL, for which the SAR procedure was designed (Galbraith et al., 1999).

The corresponding distributions of single-grain D_e_ values are shown as radial plots in Fig. S2.1. All of the D_e_ distributions are widely spread, with overdispersion values exceeding 35 ± 5% (Table S2.2). The true spread is even larger, as each of the samples contained some ‘Zero D_e_ grains’ (see Table S2.1), which we interpret as modern, intrusive grains; they have not been included in the data set of accepted grains and are not displayed in Fig. S2.1. The spread in D_e_ values for the JKF1 and JSM1 samples is much greater than can be accounted for by measurement uncertainties (and is much larger than the overdispersion value of 9 ± 4% obtained in the dose recovery test) or by differences in beta dose rate to individual grains. We attribute the wide spread in D_e_ values to post-depositional mixing of the original, ‘host’ sediments with intrusive grains that were last exposed to sunlight more recently.

For each of the JKF1 samples, we also measured 24 single aliquots of quartz (each composed of ~100 grains) using the same equipment and SAR procedures as those described by Petraglia et al. (2011) for the JQ1 samples. The D_e_ values for those aliquots that satisfied all of the reliability criteria (*n* = 15, 18 and 16 for JKF1-OSL1, JKF1-OSL3 and JKF1-OSL4, respectively) are displayed as open triangles in Fig. S2.1a–c. In each case, the range of D_e_ estimates is similar to that observed for the single grains, which demonstrates that the spread in single-grain D_e_ values is not an artefact of the specific instrumentation or measurement and data analysis procedures used. The single-aliquot D_e_ estimates for JKF1-OSL1, JKF1-OSL3 and JKF1-OSL4 are overdispersed by 42 ± 8, 28 ± 5 and 32 ± 6%, respectively: these values are slightly smaller than those of the single-grain D_e_ distributions, which is readily explained by the process of averaging that afflicts multi-grain aliquots. When a sample consists of mixed-age sediments, single-grain dating is required to resolve the separate mixing components (Arnold and Roberts, 2009; Galbraith and Roberts, 2012).

We used the finite mixture model (FMM) to identify the minimum number of discrete populations of grains needed to explain the spread in single-grain D_e_ values for each sample (Roberts et al., 2000; Galbraith and Roberts, 2012). The FMM was fitted by maximum likelihood using overdispersion values of 10–25% and optimal fits were determined from the maximum log-likelihood values and the Bayes Information Criterion (Galbraith and Roberts, 2012). The D_e_ distributions could be optimally fitted by 5, 4 or 3 discrete populations, depending on the extent of overdispersion (Table S2.2). OSL ages are listed in Table S2.2 for the two main D_e_ populations in each sample, which are highlighted by the grey bands in Fig. S2.1. In each case, these two populations account for more than three-quarters of the total number of accepted grains, whereas the minor components (grey lines in Fig. S2.1) typically represent a few grains with substantially higher or low doses.

At Jebel Katefeh, the basal sample (JKF1-OSL1) has one component with ~74% of the D_e_ values that yields a depositional age of 64 ± 6 ka (Table S2.2). This sample also contains populations of grains with much younger ages, so it has evidently not remained undisturbed since initial deposition. The same concern about post-depositional mixing also applies to the two overlying samples from artefact-bearing Stratum H. Both JKF1-OSL3 and JKF1-OSL4 were optimally fitted by 3 discrete populations of grains, in addition to the population of modern intrusive grains removed from the data set before the FMM was fitted. The two main components correspond to burial ages of ~50 ka and about 85–90 ka, but it is not obvious which of these ages relates most closely to the time of manufacture of the artefacts. These components contain an approximately equal number of grains, so there has clearly been substantial mixing of sediments after deposition. The fact that one of these components is 20–25 ka older than the majority of grains in the underlying sands might be due to these grains being incompletely bleached at the time of deposition, but this explanation seems unlikely as these samples were collected from the archaeological stratum, which implies some period of sub-aerial exposure to the intense Arabian sunlight. At present, we cannot explain this apparent anomaly, and can only bracket the ages of the Stratum H artefacts to between ~50 ka and about 85–90 ka,

At Jebel Umm Sanman, the lowermost sample (JSM1-OSL2) has two main D_e_ components, corresponding to burial ages of 61 ± 8 ka and 140 ± 14 ka (Table S2.2). Again, these two populations are present in almost equal proportions, and together comprise almost 90% of the total number of accepted grains. The two minor D_e_ components represent a total of only 4 grains with low D_e_ values (Fig. S2.1f), and only two ‘Zero D_e_ grains’ were rejected from this sample (Table S2.1), so the vast majority of grains were buried no later than ~60 ka ago and no earlier than ~140 ka ago. We consider the associated artefacts to date to within this time interval. JSM1-OSL1 has the most overdispersed D_e_ distribution of any sample examined in this study (93 ± 11%), which may be related to its proximity to the present-day ground surface. Five discrete D_e_ components were identified by the FMM, of which the main pair accounts for nearly 80% of the total number of accepted grains. These two components yield ages of 42 ± 9 ka and 96 ± 9 ka, the latter corresponding to the population of grains that comprise almost 60% of this sample (Table S2.2). Given the extent of post-depositional mixing present in both of the JSM1 samples, it is not clear which of the component ages is most appropriate for the artefacts, but we conservatively constrain their time of burial to between ~40 and ~140 ka, with the most likely interval being 60–100 ka.

To calculate all of the aforementioned OSL ages, the D_e_ values listed in Table S2.2 were divided by the corresponding total dose rates from environmental sources of ionising radiation. The latter consist mainly of beta and gamma radiation from the nuclear decay of ^238^U, ^235^U, ^232^Th (and their daughter products) and ^40^K in the deposits surrounding the dated grains, with additional contributions from cosmic rays and from alpha emitters internal to the quartz grains, which are assumed to supply 0.03 ± 0.01 Gy/ka (Table S2.2). Each of these contributions was measured or estimated in the same manner as described by Petraglia et al. (2011), including corrections to the beta dose rate for the effect of grain size and hydrofluoric acid etching, and adjustments to the beta, gamma and cosmic ray dose rates for the long-term water contents of the samples. The measured (field) water contents ranged from 0.6 to 2.7% (Table S2.2), and these were each assigned a relative uncertainty of 30% to allow for any likely variations integrated over the period of sample burial. The calculated ages are not especially sensitive to the values used, increasing (or decreasing) by ~1% for each 1% increase (or decrease) in water content.

Gamma dose rates were measured at each sample location by inserting a gamma ray detector into the holes left after removing the OSL tubes. Cosmic ray dose rates were estimated from published equations, taking into account the latitude, longitude and altitude of JKF1 and JSM1, as well as the density and thickness of sediment overburden averaged over the period since sample deposition. Beta dose rates were measured directly using a GM-25-5 low-level beta counting system, and the equilibrium status of the ^238^U and ^232^Th decay chains in three of the samples (JKF1-OSL1, JKF1-OSL3 and JSM1-OSL1) was checked by means of high-resolution gamma spectrometry (using the same instruments and calibration standards as Olley et al., 1996, 1997). The resulting radionuclide activities are listed in Table S2.3, together with the beta dose rates calculated from them.

The HRGS analyses show that the ^232^Th decay chain (represented by ^228^Ra and ^228^Th in Table S2.3) is presently in a state of secular equilibrium, as is commonly the case for terrestrial sediments. The ^238^U decay chain (represented by ^238^U, ^226^Ra and ^210^Pb) has a deficit of ^210^Pb relative to ^226^Ra in samples JKF1-OSL1 and JSM1-OSL1 (^210^Pb/^226^Ra ratios of 0.65 ± 0.12 and 0.73 ± 0.11, respectively), which we attribute to loss of radon gas to atmosphere. We have assumed that these conditions prevailed throughout the period of sample burial, but numerical modelling has shown that even the most common time-dependent disequilibria in the ^238^U series are unlikely to give rise to errors in the total dose rate of more than 2–3% when techniques such as HRGS and beta counting are employed (Olley et al., 1996, 1997). We note that the beta dose rates deduced from the HRGS measurements are systematically higher than those estimated by GM-25-5 beta counting, but the significance of these differences is questionable as the dose rates are so low (particularly that of JKF1-OSL1). For consistency, the GM-25-5 values listed in Table S2.2 were used in the total dose rate calculations for all of the JKF1 and JSM1 samples.

In conclusion, the buried artefacts at JKF1 and JSM1 can currently be constrained, with a reasonable degree of confidence, to about 50–90 ka and 60–100 ka, respectively. However, the identification of extensive post-depositional sediment mixing at both sites prohibits the determination of narrower time intervals for the stone tool assemblages.

**References**

Arnold LJ, Roberts RG (2009) Stochastic modelling of multi-grain equivalent dose (D_e_) distributions: implications for OSL dating of sediment mixtures. Quaternary Geochronology 4: 204–230.

Galbraith RF, Roberts RG (2012) Statistical aspects of equivalent dose and error calculation and display in OSL dating: an overview and some recommendations. Quaternary Geochronology 11: 1–27.

Galbraith RF, Roberts RG, Laslett GM, Yoshida H, Olley JM (1999) Optical dating of single grain and multiple grains of quartz from Jinmium rock shelter, northern Australia: Part I, experimental design and statistical models. Archaeometry 41: 339–364.

Jacobs Z, Roberts RG (2007) Advances in optically stimulated luminescence dating of individual grains of quartz from archeological deposits. Evolutionary Anthropology 16: 210–223.

Jacobs Z, Roberts RG, Galbraith RF, Deacon HJ, Grün R, et al. (2008) Ages for the Middle Stone Age of southern Africa: implications for human behavior and dispersal. Science 322: 733–735.

Jacobs Z, Meyer MC, Roberts RG, Aldeias V, Dibble H, et al. (2011) Single-grain OSL dating at La Grotte des Contrebandiers (‘Smugglers’ Cave’), Morocco: improved age constraints for the Middle Paleolithic levels. Journal of Archaeological Science 38: 3631–3643.

Jacobs Z, Roberts RG, Nespoulet R, El Hajraoui MA, Debénath A (2012) Single-grain OSL chronologies for Middle Palaeolithic deposits at El Mnasra and El Harhoura 2, Morocco: implications for Late Pleistocene human–environment interactions along the Atlantic coast of northwest Africa. Journal of Human Evolution 62: 377–394.

Jacobs Z, Hayes EH, Roberts RG, Galbraith RF, Henshilwood CS (in press) An improved OSL chronology for the Still Bay layers at Blombos Cave, South Africa: further tests of single-grain dating procedures and a re-evaluation of the timing of the Still Bay industry across southern Africa. Journal of Archaeological Science (doi:10.1016/j.jas.2012.06.037).

Olley JM, Murray A, Roberts RG (1996) The effects of disequilibria in the uranium and thorium decay chains on burial dose rates in fluvial sediments. Quaternary Science Reviews 15: 751–760.

Olley JM, Roberts RG, Murray AS (1997) Disequilibria in the uranium decay series in sedimentary deposits at Allen‘s Cave, Nullarbor Plain, Australia: implications for dose rate determinations. Radiation Measurements 27: 433–443.

Petraglia MD, Alsharekh AM, Crassard R, Drake NA, Groucutt H, et al. (2011) Middle Paleolithic occupation on a Marine Isotope Stage 5 lakeshore in the Nefud Desert, Saudi Arabia. Quaternary Science Reviews 30: 1555–1559.

Roberts RG, Galbraith RF, Yoshida H, Laslett GM, Olley JM (2000) Distinguishing dose populations in sediment mixtures: a test of single-grain optical dating procedures using mixtures of laboratory-dosed quartz. Radiation Measurements 32: 459–465.

**Table S2.1**

Numbers of grains measured, rejected and accepted for the Jebel Katefeh (JKF-1) and Jebel Umm Sanman (JSM-1) samples, and the reasons for their rejection.

Sample Grains T_N_ signal 0 Gy signal Poor No L_N_/T_N_ Depletion Zero D_e_ Grains %

code measured <3 x BG ^a^ >5% L_N_/T_N_ ^b^ recycling ^c^ intersection ^d^ by IR ^e^ grains ^f^ accepted accepted

**Jebel Katefeh**

JKF1-OSL1 3000 2639 75 117 1 120 4 44 1.5

JKF1-OSL3 4000 3636 88 97 0 115 9 55 1.4

JKF1-OSL4 2500 2274 52 56 1 67 7 43 1.7

**Jebel Umm Sanman**

JSM1-OSL1 3000 2720 46 78 2 108 6 40 1.3

JSM1-OSL2 2900 2656 58 53 9 92 2 30 1.0

^a^ Inherently dim grains: intensity of OSL signal (T_N_) induced by test dose given after measurement of natural OSL is less than 3 times the background (BG) intensity.

^b^ High recuperation: sensitivity‐corrected OSL intensity measured after 0 Gy regenerative dose is more than 5% of sensitivity-corrected natural OSL intensity (L_N_/T_N_).

^c^ Inadequate test dose sensitivity correction: recycling ratio of sensitivity-corrected OSL signals for duplicate regenerative doses differs from unity by more than 2σ.

^d^ (Over)saturated grains: sensitivity-corrected natural OSL intensity (L_N_/T_N_) equals or exceeds saturation limit of the sensitivity-corrected OSL dose response curve.

^e^ Feldspar contamination: significant loss of OSL signal upon exposure to infrared (IR) stimulation yields ratio of after/before OSL signals more than 2σ below unity.

^f^ Modern intrusive grains: grains with calculated equivalent dose (D_e_) values, including negative estimates, within measurement uncertainty of 0 Gy at 2σ.

**Table S2.2**

Dose rate data, equivalent dose (D_e_) values and OSL ages for the Jebel Katefeh (JKF-1) and Jebel Umm Sanman (JSM-1) samples.

Sample Water ———————— Dose rates (Gy/ka) ^c^ ———————— —————————— Equivalent doses ^c^ —————————— OSL age ^c^

code ^a^ (%) ^b^ beta gamma cosmic total ^d^ no. grains ^e^ OD (%) ^f^ D_e_ comp. ^g^ D_e_ (Gy) ^h^ % grains ^i^ (ka)

**Jebel Katefeh**

JKF1-OSL1 1.4 0.09 ± 0.01 0.18 ± 0.01 0.19 ± 0.02 0.49 ± 0.03 44 / 3000 62 ± 8 4 13 ± 2 20 ± 9 26 ± 5

31 ± 2 74 ± 9 64 ± 6

JKF1-OSL3 2.7 0.19 ± 0.01 0.20 ± 0.01 0.20 ± 0.02 0.62 ± 0.03 55 / 4000 35 ± 5 3 33 ± 4 57 ± 18 53 ± 6

53 ± 6 38 ± 19 86 ± 11

JKF1-OSL4 2.6 0.18 ± 0.01 0.21 ± 0.01 0.19 ± 0.02 0.61 ± 0.03 43 / 2500 43 ± 6 3 30 ± 2 41 ± 10 49 ± 5

53 ± 3 49 ± 10 87 ± 6

**Jebel Umm Sanman**

JSM1-OSL1 1.1 0.27 ± 0.02 0.40 ± 0.02 0.26 ± 0.03 0.96 ± 0.05 40 / 3000 93 ± 11 5 40 ± 9 19 ± 9 42 ± 9

92 ± 7 59 ± 10 96 ± 9

JSM1-OSL2 0.6 0.31 ± 0.02 0.41 ± 0.02 0.22 ± 0.02 0.97 ± 0.05 30 / 2900 67 ± 10 4 60 ± 7 38 ± 11 61 ± 8

136 ± 12 49 ± 11 140 ± 14

^a^ Individual grains of 180–212 μm diameter measured for all samples.

^b^ Measured (field) water contents used for dose rate and age determination and assigned relative uncertainties of 30% to accommodate (at 2σ) any likely variations in water content integrated over the period of sample burial. The calculated ages increase/decrease by about 1% for each 1% increase/decrease in water content.

^c^ Mean ± total (1σ) uncertainty, calculated as the quadratic sum of random and systematic errors.

^d^ Includes internal alpha dose rate of 0.03 ± 0.01 Gy/ka.

^e^ Number of accepted grains / number of measured grains (see Table S2.1).

^f^ Overdispersion (OD), the relative spread in the D_e_ distributions beyond that associated with the measurement uncertainties for single-grain D_e_ estimates. These estimates exclude the small number of ‘Zero D_e_ grains’ listed in Table S2.1, so the actual OD values of these samples exceed those tabulated.

^g^ Optimal number of discrete D_e_ components needed to fit the single-grain D_e_ distributions, as identified by the finite mixture model (FMM). The ‘Zero D_e_ grains’ listed in Table S2.1 were excluded from this analysis, so each sample contains an additional (0 Gy) component.

^h^ Mean ± standard error of the two major D_e_ populations (more than 75% of accepted grains) identified by the FMM. The standard error includes all measurement uncertainties, the spread due to the OD associated with the optimal model fit, and a relative error of 2% for possible bias in calibration of the laboratory beta source.

^i^ Percentage of accepted grains in each D_e_ population.

**Table S2.3**

Beta dose rates determined by high-resolution gamma-ray spectrometry (HRGS) and by GM-25-5 beta counting

| Radionuclide activities  (Bq/kg) | JKF1-OSL1 | JKF1-OSL3 | JSM1-OSL1 |
| --- | --- | --- | --- |
| ^238^U | 4.66 ± 0.69 | 10.22 ± 1.31 | 14.22 ± 1.42 |
| ^226^Ra | 5.56 ± 0.14 | 9.35 ± 0.25 | 16.23 ± 0.32 |
| ^210^Pb | 3.59 ± 0.64 | 9.51 ± 1.44 | 11.90 ± 1.74 |
| ^228^Ra | 5.10 ± 0.25 | 7.50 ± 0.46 | 16.29 ± 0.59 |
| ^228^Th | 4.85 ± 0.19 | 7.48 ± 0.31 | 15.76 ± 0.44 |
| ^40^K | 24.72 ± 1.51 | 43.69 ± 2.86 | 53.85 ± 2.99 |
| HRGS beta dose rate ^a^  (Gy/ka) | 0.12 ± 0.01 | 0.23 ± 0.02 | 0.33 ± 0.02 |
| GM-25-5 beta dose rate ^a^  (Gy/ka) | 0.09 ± 0.01 | 0.19 ± 0.01 | 0.27 ± 0.02 |
| HRGS dose rate /  GM-25-5 dose rate ^b^ | 1.37 ± 0.14 | 1.25 ± 0.12 | 1.22 ± 0.11 |

^a^ Error includes 3% systematic uncertainty assigned to beta-dose attenuation factors.

^b^ Error excludes 3% systematic uncertainty assigned to beta-dose attenuation factors.


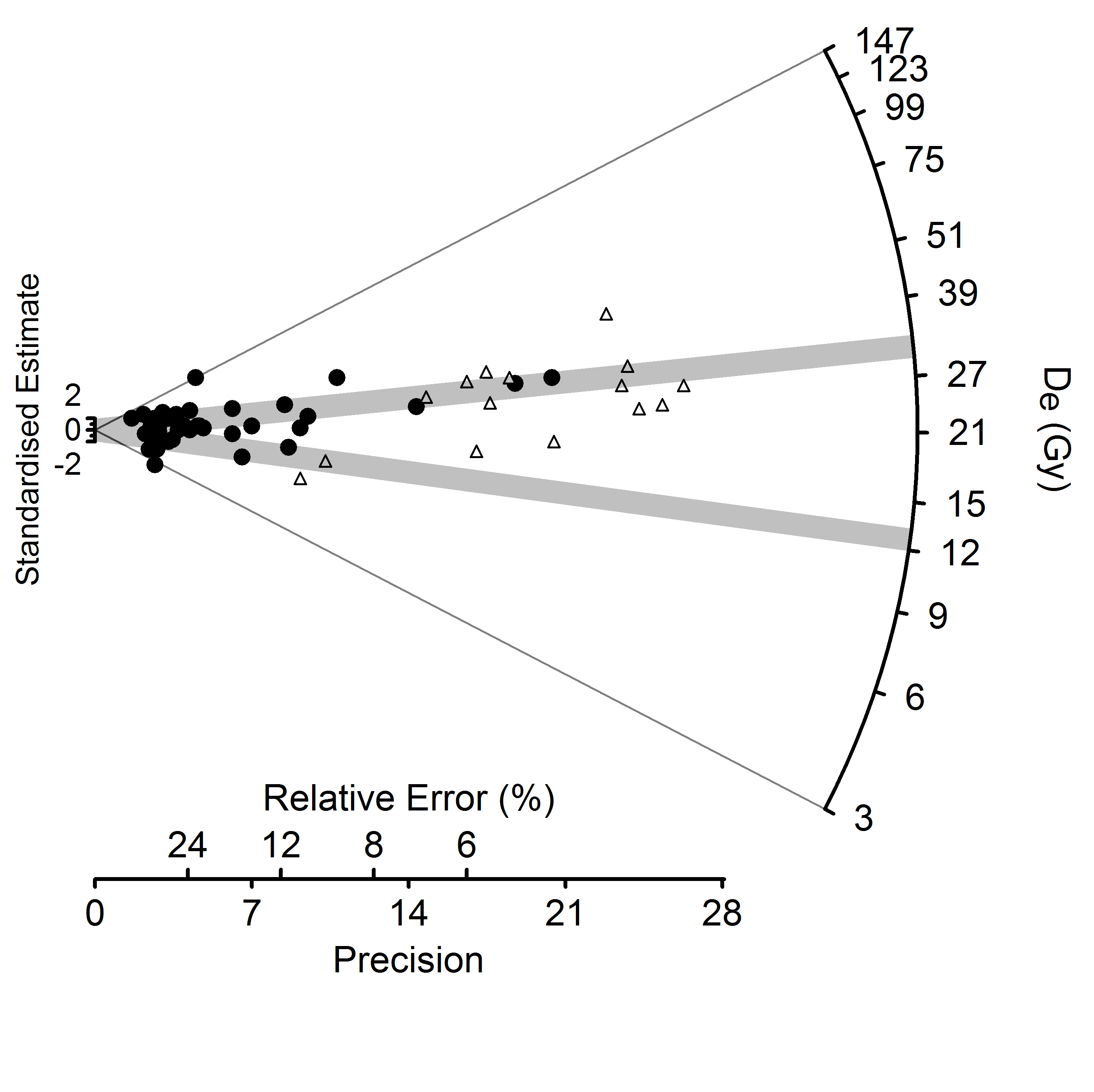

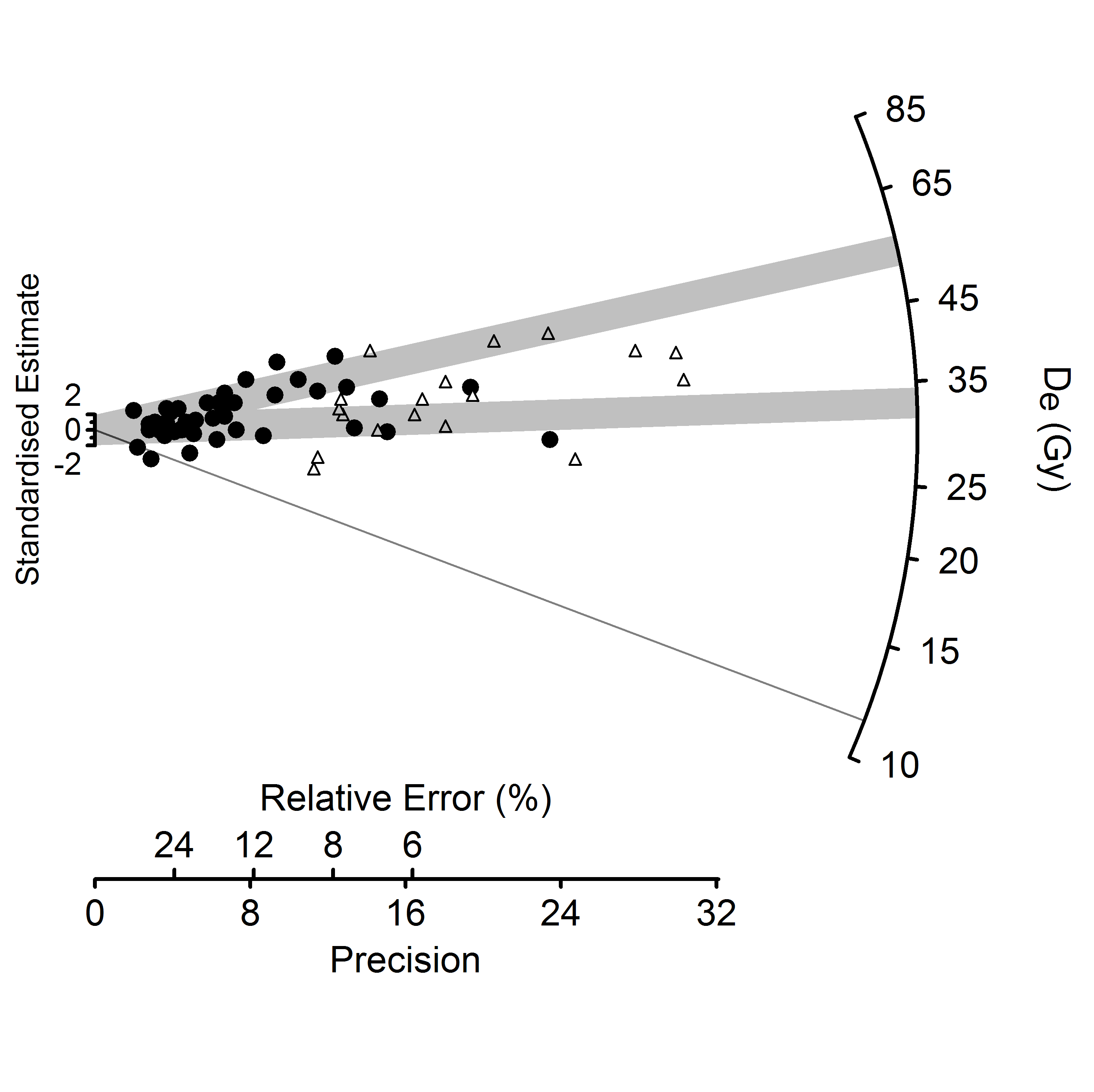

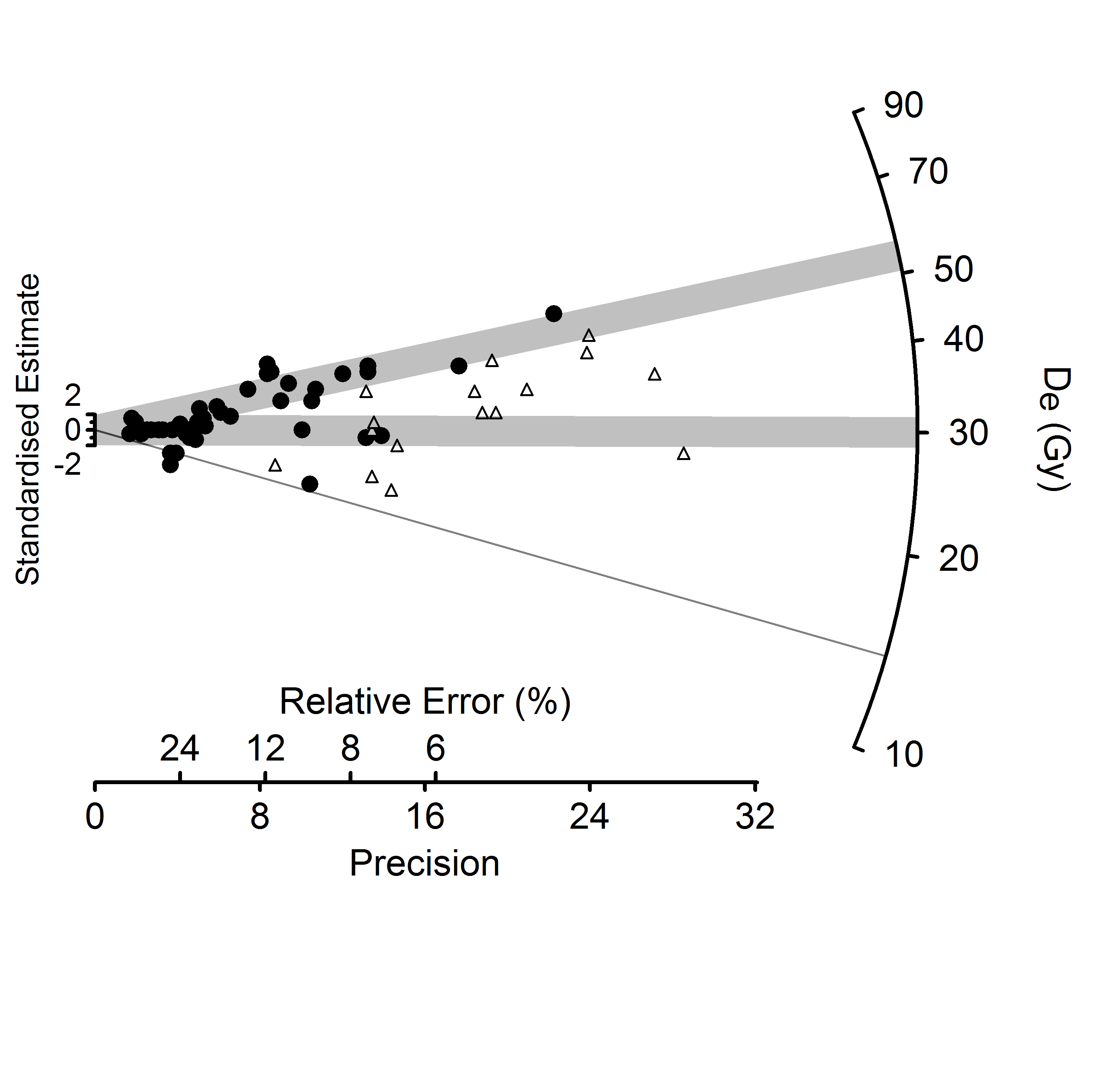

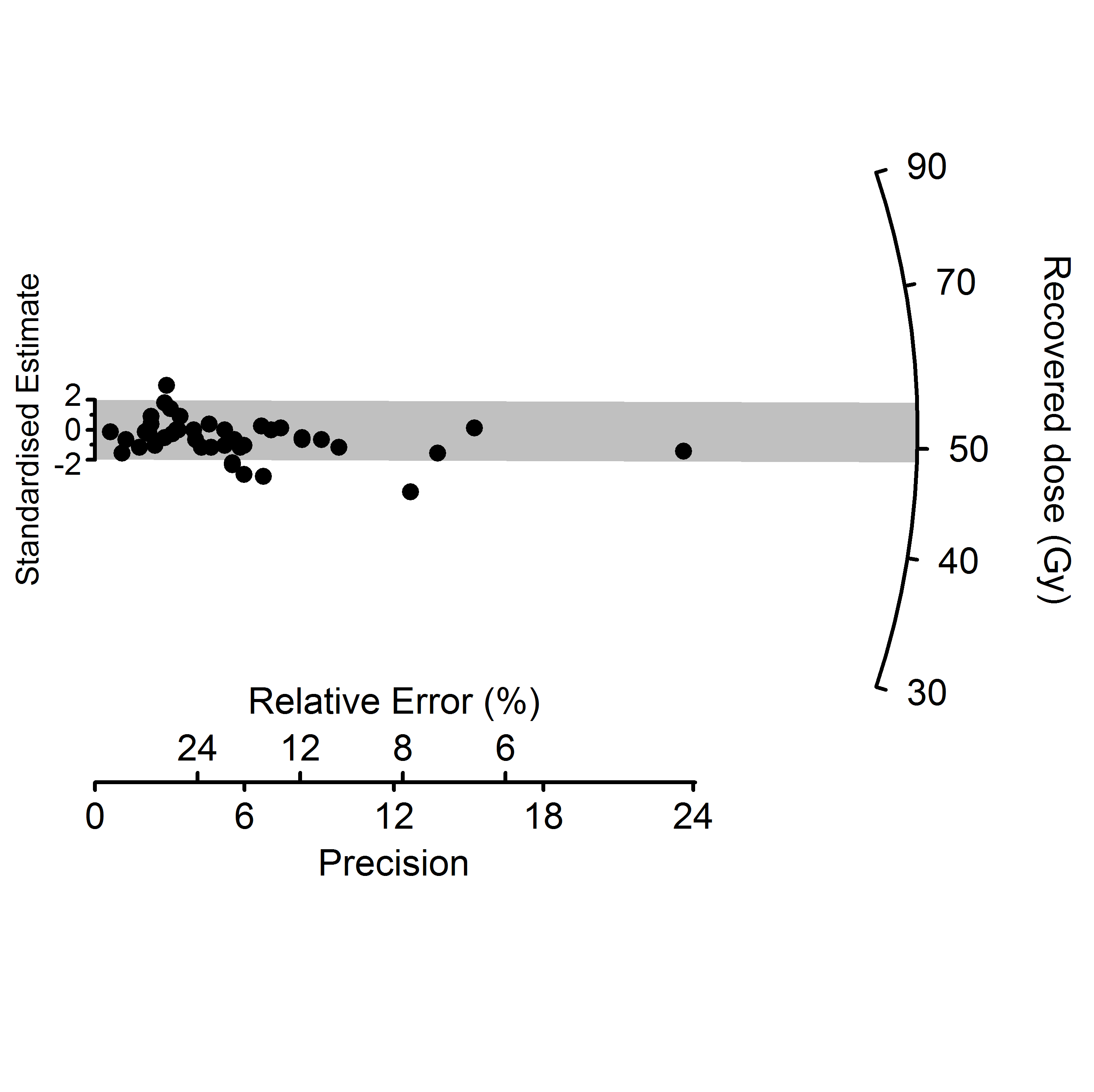


**(d) JKF1-OSL4 dose recovery**

**(a) JKF1-OSL1**

**(b) JKF1-OSL3**

**(c) JKF1-OSL4**

**(f) JSM1-OSL2**

**(e) JSM1-OSL1**


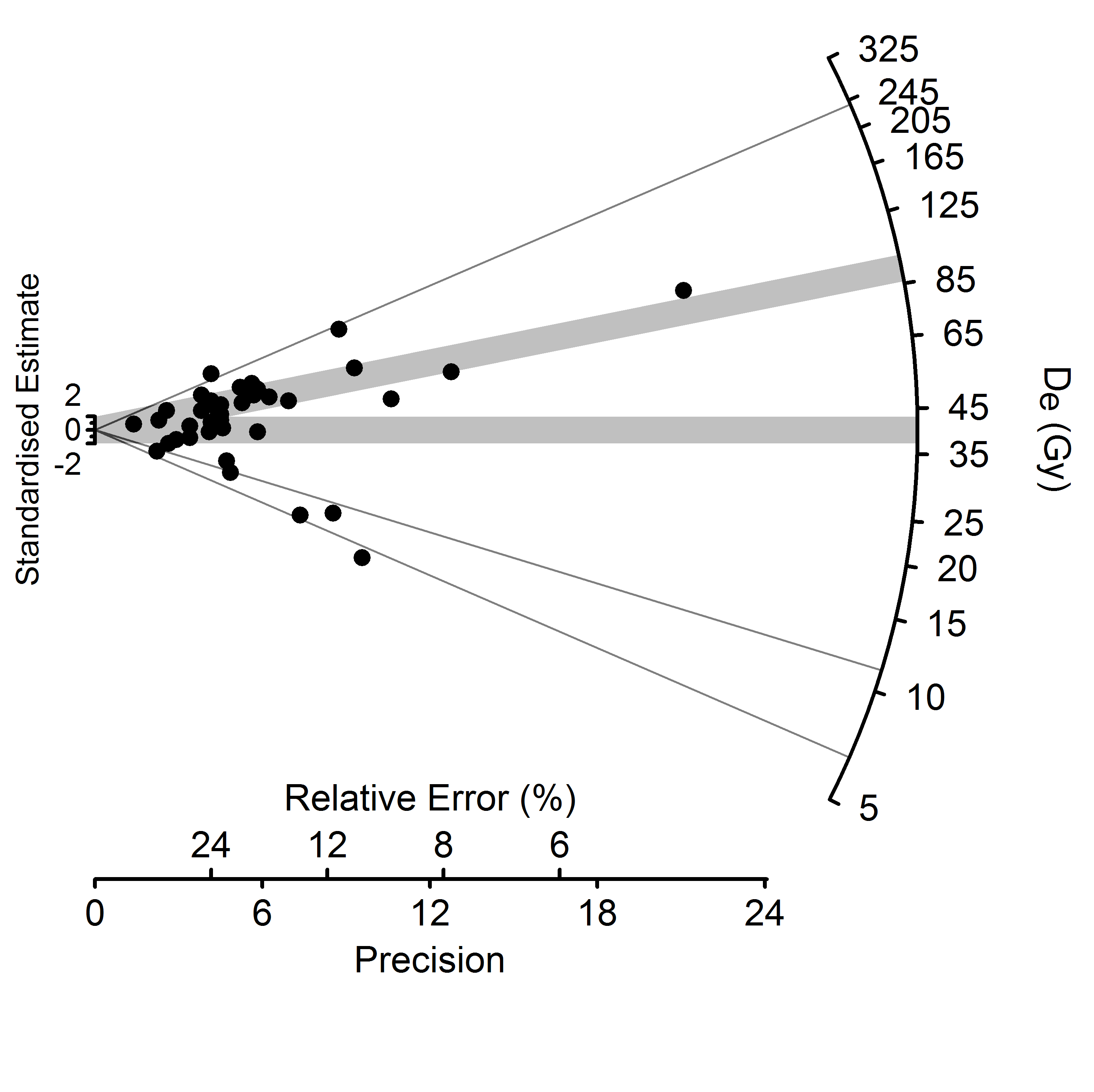

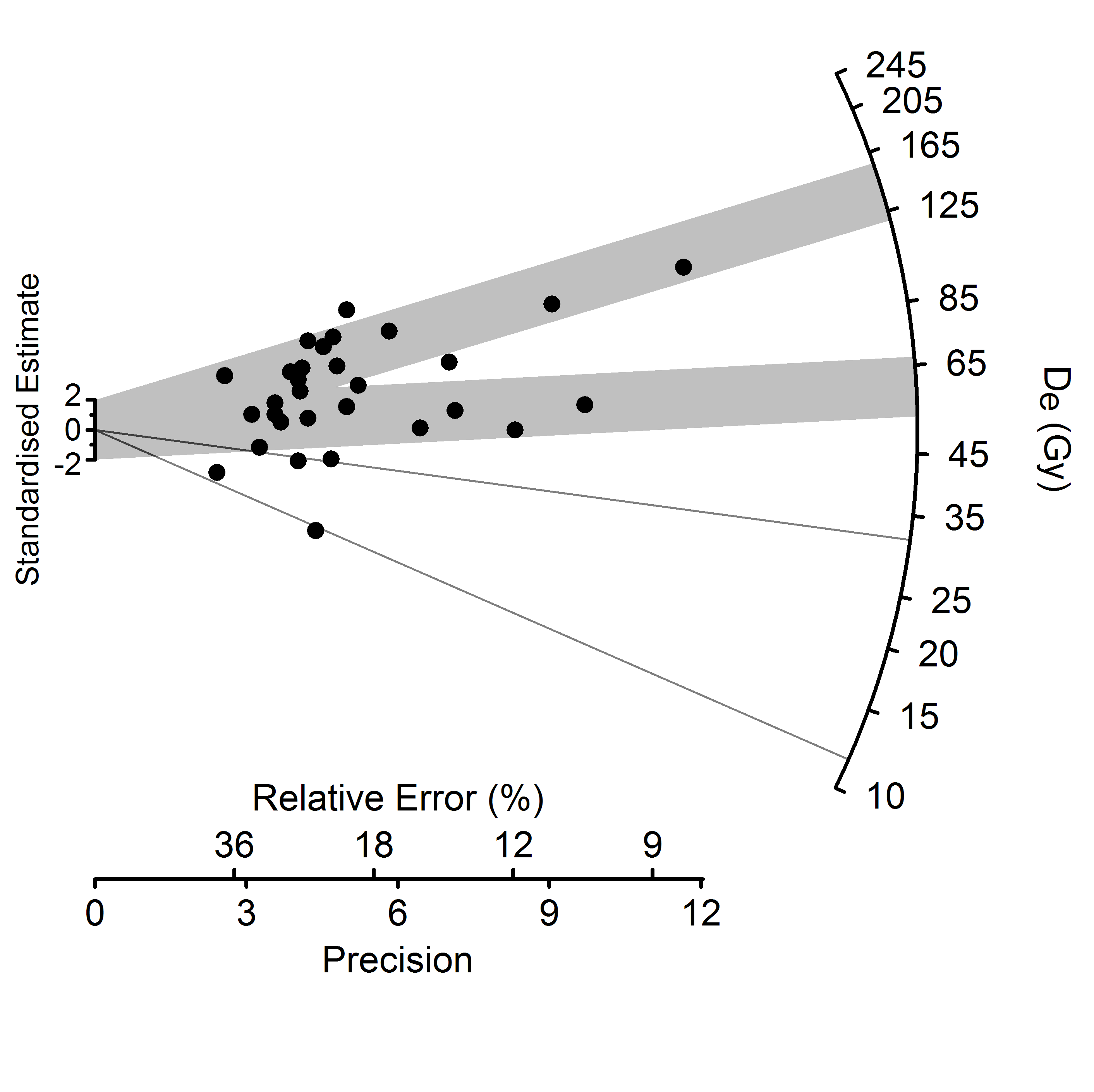


**Figure S2.1**

Radial plots of single-grain D_e_ distributions (filled circles) for the Jebel Katefeh (JKF1) and Jebel Umm Sanman (JSM1) samples. The grey bands are centred on the two main D_e_ populations in each sample (as identified by the finite mixture model), and the radiating lines indicate the other D_e_ populations fitted by the model. The open triangles in (a), (b) and (c) are the D_e_ values obtained from aliquots that each contain ~100 grains. In (d), the grey band is centred on the laboratory dose of 52 Gy given to grains of JKF1-OSL4 that had been exposed to sunlight. Measured (recovered) doses that are consistent at 2σ with this known dose will fall within the grey band, as is the case for 86% of the grains in this experiment.
