# Supplementary material for: Hominin Dispersal into the Nefud Desert and Middle Palaeolithic Settlement along the Jubbah Palaeolake, Northern Arabia
Source: PLoS One. 2012 Nov 19;7(11):e49840. doi: 10.1371/journal.pone.0049840 (PMC3501467; doi:10.1371/journal.pone.0049840)
Supplement: Supporting Information S3 — Residue analysis report. (PDF) [file pone.0049840.s003.pdf]

Supporting Information S3: Residue Analysis Report

Hominin Dispersal into the Nefud Desert and Middle Palaeolithic Settlement along the Jubbah Palaeolake, Northern Arabia

Michael D. Petraglia, Abdullah Alsharekh, Paul Breeze, Chris Clarkson, Rémy Crassard, Nick A. Drake, Huw S. Groucutt, Richard Jennings, Adrian G. Parker, Ash Parton, Richard G. Roberts, Ceri Shipton, Carney Matheson, Abdulaziz al-Omari, Margaret-Ashley Veall

The visual interpretations of the residue traces via microscopy, coupled with their observed placement and the wear patterns present on an object have been used as a proxy to determine ancient cultural practices [1-3]. Archaeological microscopy has been the traditional approach used for the analysis of residues on stone tools [4-15]. Although this method has proven to be particularly useful in identifying residues when a distinctive feature is present like hair fibres, phytoliths and pollen, a biochemical approach to residue analysis supports both the identification of contamination, as well as the authentic residue compounds (faunal or plant) that adhered to the surface of the stone artefact. The biochemical approach to archaeological residue analysis has been employed to study lipids, resins, fatty acids, waxes, oils and other compounds [16-28]

Compounds identified via a biochemical approach can be studied to determine their likely origin. In some cases these compounds will be natural compounds that are found in plant or animal sources [16-28]. However, it is also possible to identify synthetic compounds (Phthalate, Phthalic acid) that are not found in nature and their source can then be interpreted as contamination [25,29]. There are also some compounds that are found in nature and are used industrially as well (eg Pelargonic acid); hence, in this analysis we have excluded these compounds from the interpretation. It is possible to differentiate between these natural and industrial compounds based upon geography and their industrial use, however, in most cases it would not be possible to differentiate between natural source and contamination. There is also the possibility that contamination could be stratigraphically related, that is to say that the depositional layer upon which the artefacts are found, might be contaminated from the environment. An example of environmental contamination might be if two artefacts are found in the same stratigraphic layer and are characterised to have the same plant or animal biomolecules in the residue, yet the remains of this plant or animal is present within that layer. This was not observed in this analysis.

The importance of a cautious interpretation is critical in this type analysis because environmental contamination will always be present. Other forms of contamination can also be

considered a potential source for the recovered biomolecules such as handling contamination, post-excavation contamination and curatorial contamination. Many of the compounds that can be identified in an archaeological residue can also be found naturally in plant or animal materials and thus could also be considered from these sources within the environment. Hence, the cautious approach of excluding all possible contaminating sources is very critical in the interpretation of the residues.

The analytical approach for the residue analysis in this study has attempted to address a number of questions in consecutive order. 1) Is there a residue present? 2) Is it organic or inorganic? 3) Is it anthropogenic or environmental? 4) Is it plant or animal? 5) From what tissue has the residue originated? 6) What taxa has the residue originated from?

The design of this particular biochemical analysis of residues was conducted post visual inspection and preliminary microscopy which were employed to screen the artefacts for the possible presence of particles and pigments on the surface of the lithic material. If a residue was suspected then a wet removal technique was applied to the tool edge and the extracted residues were analysed. The compounds identified herein are those found in greatest abundance. This strategy is based on the assumption that the compounds found in greatest abundance will represent the majority of the constituents of the residues. This being said it should be noted that very few water soluble compounds were recovered indicating that most water soluble residue components had already been lost most probably due to taphonomy. This is a feature which we have found in other archaeological sites of similar antiquity and/or where the taphonomy suggests a degree of exposure to water. In all of these analyses there are many other compounds that have been identified however due to the low abundance they are recovered in a low concentration which makes them difficult to identify with certainty. There are also many compounds present that have yet to be identified. These are usually breakdown products of naturally occurring compounds and for that reason there are many possibilities. Although this residue analysis does not preclude the possibility of residue on the other locations of the tool, it does allow for the distinction between authentic residues and contamination.

Methods

Sampling

Low powered incident light microscopy was used to identify the presence of a residue on the selected stone tools and provide initial characterisation. Many solutions have been used for the removal of residues including water, ethanol, methanol, chloroform, acetonitrile, ethyl ether, methyl chloride, and hexanes [17,21,30-39]. Residue was removed in this study by soaking the working or platform edge of the artefact in a removal solution which consisted of a mixture of water, ethanol and acetonitrile (1:1:1). The solution and artefact were placed in a rectangular tray

with a V-shaped bottom to ensure the isolated residue removal of the sampled edge from both the dorsal and ventral sides. Samples were soaked for up to 8 hours for the removal of residue. The removal solution was chosen specifically for the analysis using absorbance spectroscopy and gas chromatography coupled mass spectrometry (GC/MS).

The advantage of analysing tool edges independently is that the analysis can isolate residues specifically associated with one edge, which provides the potential to identify tool function. A second advantage to analysing isolated tool edges can address authentication of the results. If two edges are analysed and they produce the same results then those results could be produced by contamination from the environment or depositional events. If there are similar compounds on the different edges they could be further investigated for their reliable inclusion within the interpretation but if there are different compounds on the edges then depositional environmental contamination may be able to be excluded.

Absorbance Spectroscopy

An aliquot of the removed residue in the removal solution was analysed using an Epoch™ Multi-Volume Spectrophotometer System (Biotek). A volume of 2μL was placed in duplicate on a Take 3™ plate and absorbance was measure between 200 nm and 900 nm in 5 nm increments. The data was collected and analysed using the Gen 5 software and an absorbance spectrum was generated for each of the artefact residues.

Gas Chromatography coupled Mass Spectrometry

An aliquot of the removed residue in the removal solution was placed into a sterile 2mL autosampler vial and dried under a vacuum in a freeze drier (Labconco Freezone 12) for 8 hours or until dry. The samples were then derivatised with 0.1mL of BSTFA, 1% trimethylchlorosilane (Sigma-Aldrich), and 0.9mL of cold acetonitrile (Sigma-Aldrich) [31,40]. The vials were purged with nitrogen and then sealed with teflon-coated septa. The derivatisation was completed by incubation on a Baxter Scientific Multi-Block set at 120°C for 30 minutes and subsequently cooled to room temperature.

The derivatised samples were analysed by a Varian model 450 gas chromatograph coupled with a Varian model 300-MS quadrupole GC/MS mass spectrometer equipped with factor four capillary column (VF-5ms, 30m x 0.25mm ID, DF=0.25um). Helium was used as the carrier gas at a flow rate of 1.0mL/min. Samples were introduced via splitless mode in an autosampler with the injection port at a temperature of 270°C. The column temperature was initially held at 50°C for 2 minutes then increased from 50°C to 155°C at a rate of 8°C/min and

then from 155°C to 275°C at a rate of 40°C/min and held at 275°C for 9 minutes. The ionization energy was 70eV and the ion source was set at 200°C under electron ionization (EI) conditions. The scan range was from 40 to 500m/z. The GC/MS interface temperature was set at 266°C. Output files were analysed using Varian MS workstation version 6 and the NIST98 Mass Spectral Database.

Results

The removed residue from JKF-1 Artefact 847 is consistent with the use of plant material. This is evident by the presence of fatty acids more specific with plant material. The major compounds which were recovered and identified on this artefact were phosphate, nonanedioic acid (azelaic acid), octadecanoic acid (stearic acid), hexadecanoic acid (palmitic acid), octadec-9-enoic acid (oleic acid), *cis*-13-docosenoic acid (erucic acid) and 1,2-Benzenedicarboxylic acid mono (2-ethylhexyl) ester. Some of these compounds are likely contamination and can be easily identified as such; while others, such as octadecanoic acid and hexadecanoic acid which can be found in plants, animals and human fingerprints were excluded from the analysis based on the fact that they may have resulted from post excavation contamination [41]. Cis-13-docosenoic acid, nonanedioic acid and octadec-9-enoic acid, on the other hand, are most abundant in plant sources.

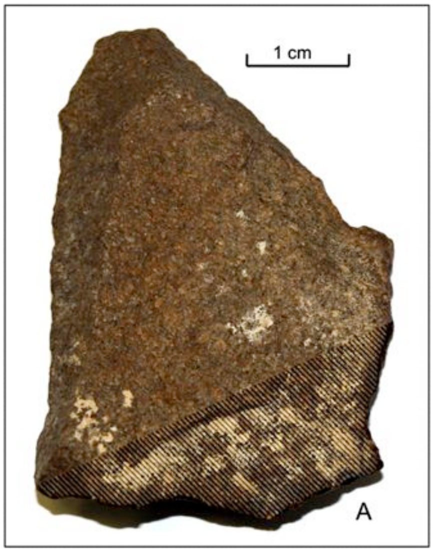

Figure S3.1. Artefact JKF-1 847, location (A) indicating where the residue has been removed for analysis.

There were two edges on JKF-1 artefact 521 where residue was removed. The material removed from location A and B had very little evidence of residue. In location A there were some minor unidentified peaks in the GCMS spectrum but the only compound found in abundance was nonanoic acid (pelargonic acid). This fatty acid is naturally found in the plants geraniums or storkbills, however, it is also used extensively for industrial uses for flavouring, plasticisers and herbicides. We have excluded this compound in our analysis and interpret it as potential contamination. We have concluded that any residue present on this artefact is too low in concentration for detection or there is no authentic residue present.

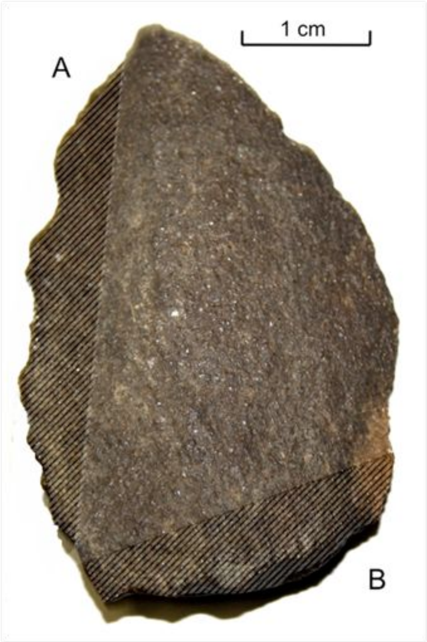

Figure S3.2. Artefact JKF-1 521, (A) and (B) indicate the locations where the residue was removed for further analysis.

There were two edges on JKF-1 artefact 504 that were analysed. If there was a residue in location A the biomolecules present were found to be undetectable. However in location B there were compounds that indicated both plant and animal residues were present. There were modified carbohydrates and other compounds but the majority of compounds recovered which

indicate plant and animal sources were fatty acids. The compounds found in greatest abundance included phosphate, glycerol, nananoic acid (pelargonic acid), tetradecanoic acid (myristic acid), pentadecanoic acid (Pentadecylic acid), *trans*-9-hexadecenoic acid (palmitelaidic acid), hexadecanoic acid (palmitic acid), trans-9-octadecanoic acid (elaidic acid), octadecanoic acid (stearic acid), furanosides and pyranosides. Nananoic acid, hexadecanoic acid and octadecanoic acid can be interpreted as contamination and were therefore excluded from the analysis. Some of these compounds like *trans*-9-octadecanoic acid and tetradecanoic acid can be found in plant or animal sources while others such as pentadecanoic acid are found in greatest abundance in animal sources especially in fatty tissues. The compound *trans*-9-hexadecenoic acid can be found in plant oils and the carbohydrates listed above are found in plant material in great abundance.

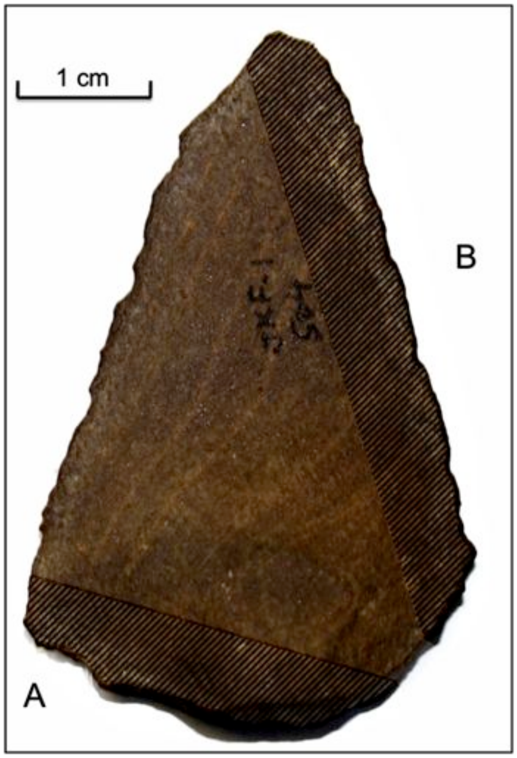

Figure S3.3. Artefact JKF-1 504, (A) and (B) indicate the two locations where residue was removed for further analysis.

There were very few biomolecules recovered from artefact 209. The compounds recovered include glycerol, urea and hexadecanoic acid. Urea can be a breakdown product of protein but can also be found as contamination. Likewise the hexadecanoic acid can be contamination from handling or even environmental contamination.

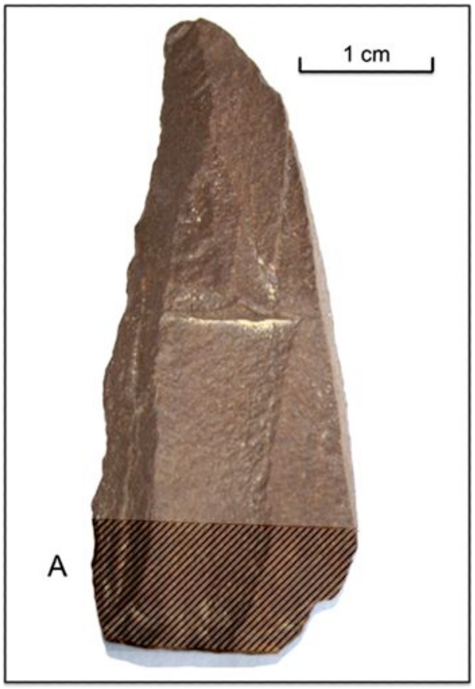

Figure S3.4. Artefact JKF-1 209, location (A) indicating where the residue has been removed for analysis.

The platform of artefact 873 was analysed (Figure S3.5). The compounds identified in this residue are consistent with animal material. There was glycerol, hexadecanoic acid (palmitic acid), *trans*-9-octadecanoic acid (elaidic acid), hexanedioic acid (adipic acid) and octadecanoic acid (stearic acid) identified within the residue in greatest abundance. Hexanedioic acid is found with the oxidation of fats predominantly with animal fats. *Trans*-9-octadecanoic acid, hexadecanoic acid, octadecanoic acid and glycerol can be found in both plant and animal while hexadecanoic acid, octadecanoic acid and glycerol can also be from environmental or handling contamination. The presence of authentic animal residue on the platform of the artefact would suggest that residues have been pushed to this area of the tool as the result of repeated use.

Presently, there is no indication of the presence of resin acids which would be indicative of the use of resin as a binding material for tool hafting.

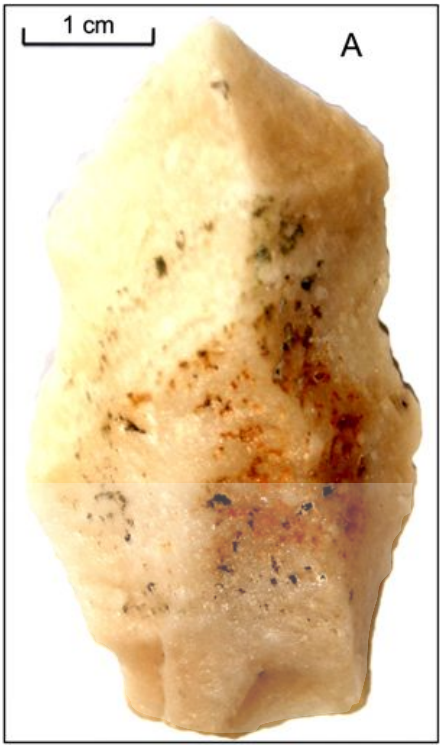

Figure S3.5. Artefact JKF-1 873, location (A) indicating where the residue has been removed for analysis.

Analysis of the residue from artefact S-76 was from one location. This artefact was abundant in residue and likewise the range of compounds recovered was large. The compounds recovered in greatest abundance and identified from artefact S-76 include urea, glycerol, nonanoic acid (pelargonic acid), dodecanoic acid (lauric acid), tetradecanoic acid (myristic acid), pentadecanoic acid (Pentadecylic acid), hexadecanoic acid (palmitic acid), *trans*-9-hexadecenoic acid (palmitelaidic acid), octadec-9-enoic acid (oleic acid), octadecanoic acid (stearic acid), pyranosides and furanosides. Many of these compounds can be found in plants and animals and are thus difficult to interpret when identifying the source of the residue. In this instance it is most likely that multiple residues are present on this artefact along with environmental and handling contamination. Dodecanoic acid is found predominantly in plant sources and usually plants with herbal properties but can also be found in some animal materials especially milk. Tetradecanoic

acid is predominantly found in plant oils but can also be found in animal fats. Pentadecanoic and *trans*-9-hexadecenoic acids are predominantly found in animal sources particularly animal fats and milk. Although the compounds found in greatest abundance are consistent with the residue originating from plant, the possibility of secondary use on animal is highly probable.

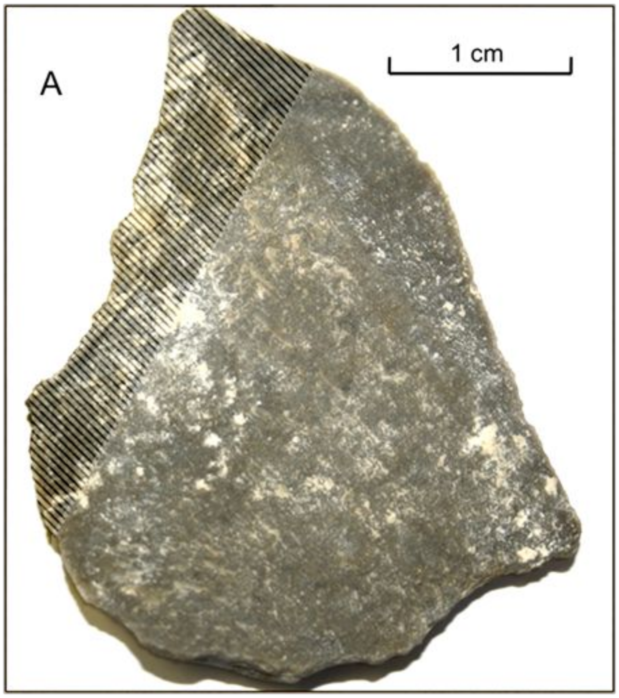

Figure S3.6. Artefact JKF-1 S-76, location (A) indicating where the residue has been removed for analysis.

The residue was removed from just one edge of artefact S-20. The compounds of greatest abundance support an interpretation that this artefact was used on plant material. These compounds include urea, glycerol, butanedioic acid (succinic acid), nonanoic acid (pelargonic acid), dodecanoic acid (lauric acid), hexadecanoic acid (palmitic acid), octadecanoic acid (stearic acid), *cis*-13-docosenoic acid (erucic acid, sterols, pyranoses, furanoses and 3-cyclohexylidene-5-(4-octadecanoyloxyphenyl)-3H-Furan-2-one. All of the above listed compounds can be found in plant sources; however, nonanoic acid, hexadecanoic acid and octadecanoic acid were excluded from the analysis due to their origin possibly being from contamination.

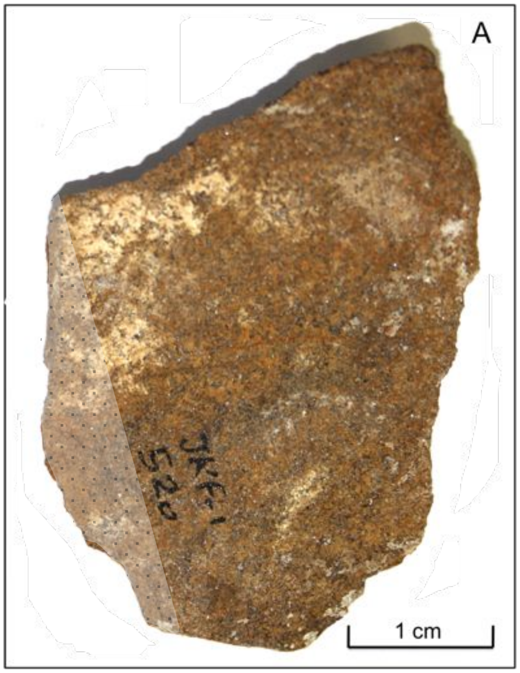

Figure S3.7. Artefact JKF-1 S-20, location (A) indicating where the residue has been removed for analysis.

Table S3.1. Summary of biochemical results for the analysis of the artefacts

Artefact	Residue	Organic	Anthropogenic	Residue Analysis Results	Contamination
S-76	YES	YES	YES	abundant evidence of plant (fatty acids, hydrocarbons, sterols) and animal (fatty acids)	fatty acids
504	YES	YES	YES	evidence of plant and animal (fatty acids)	fatty acids
S-20	YES	YES	YES	abundant evidence of plant - Brassicaceae (fatty acids, hydrocarbons, sterols)	fatty acids, hydrocarbons
847	YES	YES	YES	evidence of plant (Brassicaceae)	fatty acids
521	YES	YES	NO	No evidence of authentic residue	fatty acids
209	YES	YES	Inderteminate	little evidence (Urea - protein breakdown or urine)	fatty acids
873	YES	YES	YES	evidence of animal (fatty acids)	Fatty acids

Discussion

Biochemical analysis of archaeological residues relies on the identification of specific biomolecules. The source of these biomolecules must be interpreted within the context of the site, as well as the anthroprogenic and environmental influences that may have impacted the site over time. While some biomolecules can be found to be unique, other compounds are almost ubiquitous. In this analysis a large variety of compounds were identified (fatty acids, sterols, carbohydrates, urea, glycerol, phosphate) from the residues of the artefacts. Although some of these compounds were of plant and animal origin others can be interpreted as breakdown products and contamination [38,39,42]. There are some compounds that are found in both plant and animal sources yet may be interpreted as contamination. The most notable of these compounds include hexadecanoic acid (Palmitic acid) and octadecanoic acid (Stearic acid), the two highest concentrations of fatty acids in human fingerprints [41]. Some of the fatty acids identified include succinic acid, pelargonic acid, lauric acid, Urucic acid, palmitic acid, stearic acid, myristic acid, palmitelaidic acid, oleic acid, azelaic acid and elaidic acid. Plant residues were interpreted from the molecules on two of the seven artefacts (847 and S-20), animal residue was interpreted from one of the seven artefacts (873), evidence of a highly degraded residue or no residue was found on two of the artefacts (209 and 521) while contamination was found on all seven artefacts. Two of the seven artefacts (504 and S-76) were indeterminate and could be a mixture with a combination of plant, animal or contamination identified. Plastics, including plasticisers, were also randomly found on the artefacts however it is believed these contaminated the artefacts either in from the environment or during the curation of the artefacts in plastic bags [29]. It should be noted that in this analysis there were no two artefacts that had the same type of residue suggesting that these residues are authentic. If there was environmental contamination then these artefacts would be covered with the residue from the environmental contamination. There was no support for the identification of tissue type or taxa for the plant or animal of origin for any of the residues. Most of the biomolecules were not specific to particular tissues or taxa.

References

1. Anderson PC (1980) A Testimony of Prehistoric Tasks: Diagnostic Residues on Stone Tool Working Edges. World Archaeology 12: 181-194.
2. Robertson G (2002) Birds of a Feather Stick: Microscopic Feather Residues on Stone Artefacts from Deep Creek Shelter, New South Wales. Tempus 7: 175-182.
3. Fullagar R, Field J, Denham T, Lentfer C (2006) Early and mid Holocene tool-use and processing of taro (Colocasia esculenta), yam (Dioscorea sp.) and other plants at Kuk Swamp in the highlands of Papua New Guinea. Journal of Archaeological Science 33: 595-614.
4. Williamson B (1996) Preliminary Stone Tool Residue Analysis from Rose Cottage Cave. Southern African Field Archaeology 5: 36-44.
5. Loy TH (1993) The artifact as site; an example of the biomolecular analysis of organic residues on prehistoric tools. World Archaeology 25: 44-62.
6. Hall J, Higgins S, Fullagar R (1989) Plant residues on stone tools. In: Beck W, Clarke AF, Head L, editors. Plants in Australian archaeology. St. Lucia, Qld.: Anthropology Museum University of Queensland. pp. viii, 213.
7. Hardy BL, Kay M, Marks AE, Monigal K (2001) Stone tool function at the paleolithic sites of Starosele and Buran Kaya III, Crimea: behavioral implications. Proceedings of the National Academy of Sciences of the United States of America 98: 10972-10977.
8. Sobolik KD (1996) Lithic Organic Residue Analysis: An Example from the Southwestern Archaic. Journal of Field Archaeology 23: 461-469.
9. Rots V, Williamson BS (2004) Microwear and residue analyses in perspective: the contribution of ethnoarchaeological evidence. Journal of Archaeological Science 31: 1287-1299.
10. Barton H (1990) Raw Material and Tool Function: A residue and use-wear analysis of artefacts from a Melanesian rock shelter [BA(Hon)]. Sydney: University of Sydney.
11. Lombard M (2008) Finding resolution for the Howiesons Poort through the microscope: micro-residue analysis of segments from Sibudu Cave, South Africa. Journal of Archaeological Science 35: 26-41.
12. Lombard M (2011) Quartz-tipped arrows older than 60 ka: further use-trace evidence from Sibudu, KwaZulu-Natal, South Africa. Journal of Archaeological Science 38: 1918-1930.
13. Lombard M, Wadley L, Jacobs Z, Mohapi M, Roberts RG (2010) Still Bay and serrated points from Umhlatuzana Rock Shelter, Kwazulu-Natal, South Africa. Journal of Archaeological Science 37: 1773-1784.
14. Fullagar R, Furby J, Hardy BL (1996) Residues on stone artefacts : state of a scientific art. Antiquity 70: 740-745.
15. Hardy BL, Garufi GT (1998) Identification of Woodworking on Stone Tools through Residue and Use-Wear Analyses : Experimental Results. Journal of Archaeological Science 25: 177-184.
16. Evershed RP (1993) Biomolecular archaeology and lipids. World Archaeology 25: 74-93.
17. Ribechini E, Modugno F, Colombini MP, Evershed RP (2008) Gas chromatographic and mass spectrometric investigations of organic residues from Roman glass unguentaria. J Chromatogr A 1183: 158-169.

18. Romanus K, Baeten J, Poblome J, Accardo S, Degryse P, et al. (2009) Wine and olive oil permeation in pitched and non-pitched ceramics: relation with results from archaeological amphorae from Sagalassos, Turkey. Journal of Archaeological Science 36: 900-909.
19. Evershed RP, Dudd SN, Anderson-Stojanovic VR, Gebhard ER (2003) New Chemical Evidence for the Use of Combed Ware Pottery Vessels as Beehives in Ancient Greece. Journal of Archaeological Science 30: 1-12.
20. Rosen B, Galili E, Sharvit J (2001) Traces of Fatty Acids from a Byzantine Sounding Lead Recovered off the Israeli Coast. Journal of Archaeological Science 28: 1323-1327.
21. Modugno F, Ribechini E, Colombini MP (2006) Aromatic resin characterisation by gas chromatography-mass spectrometry. Raw and archaeological materials. Journal of Chromatography A 1134: 298-304.
22. Modugno F, Ribechini E, Colombini MP (2006) Chemical study of triterpenoid resinous materials in archaeological findings by means of direct exposure electron ionisation mass spectrometry and gas chromatography/mass spectrometry. Rapid communications in mass spectrometry 20: 1787-1800.
23. Mizzoni F, Cesaro SN (2007) Study of the organic residue from a 2600-year old Etruscan plumpekanne. Spectrochim Acta A Mol Biomol Spectrosc 68: 377-381.
24. Patrick M, Koning AJ, Smith AB (1985) Gas Liquid Chromatographic Analysis of Fatty Acids in Food Residues from Ceramics Found in the Southwestern Cape, South Africa. Archaeometry 27: 231-236.
25. d'Errico F, Backwell L, Villa P, Degano I, Lucejko JJ, et al. (2012) Early evidence of San material culture represented by organic artifacts from Border Cave, South Africa. Proc Natl Acad Sci U S A.
26. Villa P, Soriano S, Tsanova T, Degano I, Higham TF, et al. (2012) Border Cave and the beginning of the Later Stone Age in South Africa. Proc Natl Acad Sci U S A.
27. Regert M, Delacotte J-M, Menu M, Petrequin P, Rolando C (1998) Identification of Neolithic Hafting Adhesives From Two Lake Dwellings at Chalain (Jura, France). Ancient Biomolecules 2: 81-96.
28. Regert M, Vacher S, Moulherat C, Decavallas O (2003) Adhesive Production and Pottery Function During the Iron Age at the Site of Grand Aunay (Sarthe, France)*. Archaeometry 45: 101-120.
29. Wormuth M, Scheringer M, Vollenweider M, Hungerbuhler K (2006) What are the sources of exposure to eight frequently used phthalic acid esters in Europeans? Risk Anal 26: 803-824.
30. Rafferty SM (2006) Evidence of early tobacco in Northeastern North America? Journal of Archaeological Science 33: 453-458.
31. Barnard H, Ambrose SH, Beehr DE, Forster MD, Lanehart RE, et al. (2007) Mixed results of seven methods for organic residue analysis applied to one vessel with the residue of a known foodstuff. Journal of Archaeological Science 34: 28-37.
32. Copley MS, Berstan R, Dudd SN, Straker V, Payne S, et al. (2005) Dairying in antiquity. I. Evidence from absorbed lipid residues dating to the British Iron Age. Journal of Archaeological Science 32: 485-503.
33. Grimalt JO, Simoneit BRT, Hatcher PG, Nissenbaum A (1988) The molecular composition of ambers. Advances in Organic Geochemistry 13: 677-690.

34. Domenech-Carbo MT, Osete-Cortina L, de la Cruz Canizares J, Bolivar-Galiano F, Romero-Noguera J, et al. (2006) Study of the microbiodegradation of terpenoid resin-based varnishes from easel painting using pyrolysis-gas chromatography-mass spectrometry and gas chromatography-mass spectrometry. Analytical and Bioanalytical Chemistry 385: 1265-1280.
35. Mathe C, Connan J, Archier P, Mouton M, Vieillescazes C (2007) Analysis of frankincense in archaeological samples by gas chromatography-mass spectrometry. Ann Chim 97: 433-445.
36. Pitthard V, Griesser M, Stanek S, Bayerova T (2006) Study of Complex Organic Binding Media Systems on Artworks Applying GC-MS Analysis: Selected Examples from the Kunsthistorisches Museum, Vienna. Macromolecular Symposia 238: 37-45.
37. Colombini MP, Modugno F, Silvano F, Onor M (2000) Characterization of the Balm of an Egyptian Mummy from the Seventh Century B.C. Studies in Conservation 45: 19-29.
38. Colombini MP, Modugno F, Ribechini E (2005) Organic mass spectrometry in archaeology: evidence for Brassicaceae seed oil in Egyptian ceramic lamps. J Mass Spectrom 40: 890-898.
39. Regert M, Colinart S, Degrand L, Decavallas O (2001) Chemical Alteration and Use of Beeswax Through Time: Accelerated Ageing Tests and Analysis of Archaeological Samples from Various Environmental Contexts. Archaeometry 43: 549-569.
40. Shen X, Deng C, Wang B, Dong L (2006) Quantification of trimethylsilyl derivatives of amino acid disease biomarkers in neonatal blood samples by gas chromatography-mass spectrometry. Analytical and Bioanalytical Chemistry 384: 931-938.
41. Croxton RS, Baron MG, Butler D, Kent T, Sears VG (2010) Variation in amino acid and lipid composition of latent fingerprints. Forensic Science International 199: 93-102.
42. Colombini MP, Giachi G, Modugno F, Ribechini E (2005) Characterisation of organic residues in pottery vessels of the Roman age from Antinoe (Egypt). Microchemical Journal 79: 83-90.
