# Supplementary material for: Hominin Dispersal into the Nefud Desert and Middle Palaeolithic Settlement along the Jubbah Palaeolake, Northern Arabia
Source: PLoS One. 2012 Nov 19;7(11):e49840. doi: 10.1371/journal.pone.0049840 (PMC3501467; doi:10.1371/journal.pone.0049840)
Supplement: Supporting Information S4 — Core analysis report. (DOCX) [file pone.0049840.s004.docx]

## Supporting Information S4: Core Analysis Report

**Hominin Dispersal into the Nefud Desert and Middle Palaeolithic Settlement along the Jubbah Palaeolake, Northern Arabia**

**Michael D. Petraglia, Abdullah Alsharekh, Paul Breeze, Chris Clarkson, Rémy Crassard, Nick A. Drake, Huw S. Groucutt, Richard Jennings, Adrian G. Parker, Ash Parton, Richard G. Roberts, Ceri Shipton, Carney Matheson, Abdulaziz al-Omari, Margaret-Ashley Veall**

To situate the technology used at the Jubbah sites in a wider context, the 55 cores from Jebel Qattar-1 and Jebel Katefeh-1 were compared to those from neighbouring regions. The comparative sample included Middle Stone Age cores from the Horn of Africa (N=28); cores from the Levantine Mousterian sites of Tabun Cave layer C (N=13) and El Wad (N=39); cores from the early *H. sapiens* site of Skhul in the Levant (N=35); cores from Aterian sites in North Africa (N=23); cores from the Middle Palaeolithic levels of the Haua Fteah in Libya (N=41); and cores from both above (N=10) and below (N=18) the Toba ash in India. To assess which cores the Jubbah ones were most similar to, a Discriminant Functions Analysis was performed on the following set of quantitative technological variables: the proportion of blade scars, the platform angle, the relative height of intersection between the hemispheres of the cores, the scar pattern index [1], the distal curvature of the upper hemisphere, the elongation of the cores and their relative thickness. Several variables required transformations to give a normal distribution. The first Discriminant Function accounted for 32% of the variance and the second accounted for 25.4%. Wilks’ lambda values of 0.353 and 0.484 were both significant at the P<0.001 level, indicating much of the variability in these functions could be attributed to between rather than within group differences. Figure 17 shows the group centroids for the different core groups. The structure matrix (Table 7) indicates that high values of function 1 represent a high proportion of blades, high platform angles, relatively thick cores, a low scar pattern index (more parallel flaking) and a high point of intersection between core hemispheres. High values of function 2 also indicate high proportions of blades and parallel flaking, but for elongate cores with low platform angles. Numerous different analyses were run eliminating and adding different variables depending on their strength and technological relevance and the one chosen here reflects the most consistent patterning of the group centroids. The Aterian cores always associate with those from the Haua Fteah, which is perhaps not surprising given that both assemblages are from North Africa and associated with *H. sapiens*. The Skhul cores also were usually found in this group although sometimes they were associated with the Horn of Africa and El Wad cores. The Horn of Africa and El Wad cores typically have low values for functions 1 and 2 and may be characterised as relatively undifferentiated Middle Palaeolithic assemblages. The Indian cores are similar to each other above and below the Toba ash as has been found in previous studies [2], [3] and were not particularly similar to any other core groups in the analysis. The Jubbah sites are most similar to each other and interestingly always cluster with those from Tabun layer C. These three assemblages are largely differentiated by high values for function 2 so they may be characterised as being elongate with parallel flaking and low platform angles.

**References**

1. Clarkson C, Vinicius L, Lahr MM (2006) Quantifying flake scar patterning on cores using 3D recording techniques J Arch Sci 33: 132-136.

2. Petraglia MD, Korisettar R. Boivin N, Clarkson C, Ditchfield P, et al. (2007) Middle Paleolithic assemblages from the Indian subcontinent before and after the Toba super-eruption. Science 317: 114-116.

3. Clarkson C, Jones S, Harris C (2012) Continuity and change in the lithic industries of the Jurreru Valley, India, before and after the Toba eruption. Quat Internat 258: 165-179.
